# Type I IFN dynamics orchestrate protective ICAM1^+^ neutrophil heterogeneity as an early checkpoint for survival in lethal viral infection

**DOI:** 10.1016/j.isci.2026.117189

**Published:** 2026-08-18

**Authors:** Riho Saito, Yuan Ding, Kureha Satou, Fumi Yamamoto, Akisawa Satomi, Hiroki Sugishita, Hideki Ukai, Yukiko Gotoh, Tomohiko Okazaki

**Affiliations:** 1 Institute for Genetic Medicine, Hokkaido University, Sapporo, Hokkaido 060-0815, Japan; 2 Laboratory of Molecular Biology, Graduate School of Pharmaceutical Sciences, The University of Tokyo, Tokyo 113-0033, Japan; 3 ES-mouse/Virus Core, International Research Center for Neurointelligence (WPI-IRCN), UTIAS, The University of Tokyo, Tokyo 113-0033, Japan; 4 International Research Center for Neurointelligence (WPI-IRCN), The University of Tokyo, Tokyo 113-0033, Japan; 5 Japan Science and Technology Agency (JST) Fusion Oriented Research for Disruptive Science and Technology (FOREST) Program, Tokyo 102-0074, Japan; 6 Suntory SunRiSE Fellow, Suntory Foundation of Life Sciences, Kyoto 619-0284, Japan

**Keywords:** viral infection, VSV, innate immunity, neutrophil, type I IFN

## Abstract

The early host response influences outcomes following viral infection. Here, we leveraged variability in survival after intranasal vesicular stomatitis virus (VSV) challenge in genetically identical mice to retrospectively profile systemic immune responses associated with survival or lethality. Survival was strongly associated with a robust systemic type I interferon (IFN) surge within 24 h of infection, and blockade of type I IFN signaling during this narrow early window markedly reduced survival, establishing early IFN induction as a key determinant of outcome. This protective IFN surge rapidly remodeled the systemic immune landscape, inducing a specialized ICAM1^+^ neutrophil subset, primed within the bone marrow and characterized by a pro-inflammatory signature and enhanced phagocytic activity. Importantly, neutrophil-specific deletion of ICAM1 significantly reduced survival following viral infection. Together, these findings identify an early type I IFN-dependent checkpoint that shapes protective ICAM1^+^ neutrophil heterogeneity and disease trajectories, with implications for prognostic strategies and host-directed therapies.

## Highlights

• Early systemic IFN responses determine survival after VSV infection
• Survival-associated IFN surge correlates with ICAM1^+^ neutrophil polarization
• ICAM1^+^ neutrophils exhibit pro-inflammatory and enhanced phagocytic features
• Neutrophil-specific ICAM1 deletion impairs antiviral host protection

## Graphical abstract

**Figure:**
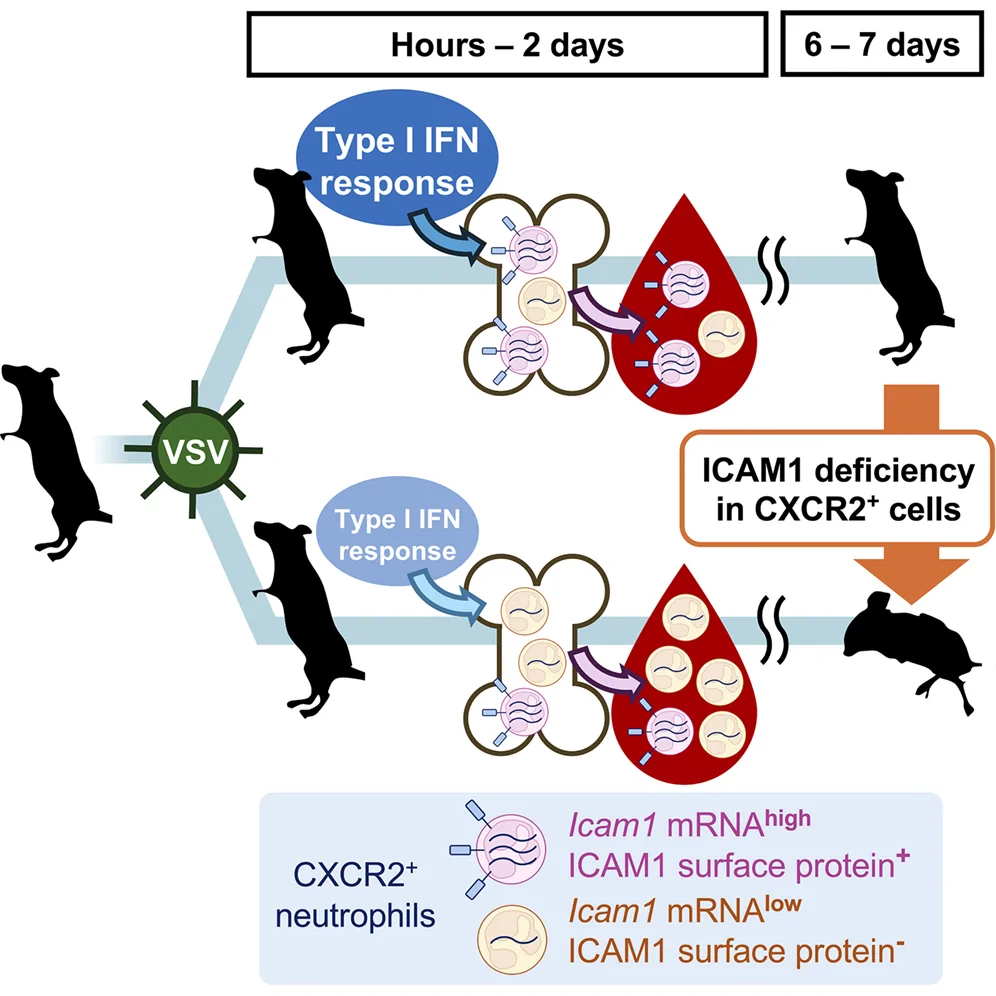

## Teaser

Biological sciences; Immunology

## Introduction

The severity of viral infections varies considerably among individuals, a phenomenon arising from the complex interplay among host, viral, and environmental factors.^1^ Despite a growing understanding of risk factors, elucidating the precise mechanisms that determine the ultimate clinical outcome remains a major challenge. Addressing this challenge is critical for the early prediction of individual outcomes, thereby enabling timely therapeutic intervention.

Previous studies have tackled this challenge by comparing immune cell and cytokine profiles in patients with mild versus severe outcomes. These studies have provided important insights, revealing associations between myeloid dysregulation, lymphoid impairment, or aberrant cytokine responses and disease severity in infections such as SARS-CoV-2^2^^,^^3^^,^^4^^,^^5^ and influenza virus.^6^ However, they are limited in their ability to elucidate the crucial early events of infection, as samples are typically collected only after symptoms have become apparent. Human challenge studies can probe the initial phase^7^^,^^8^ but are ethically restricted to inducing only mild outcomes, leaving the initial host responses that distinguish mild from severe outcomes largely uncharacterized.

Animal models are indispensable for dissecting these early, dynamic events. Intriguingly, significant variability in disease outcome often emerges even in highly controlled experimental settings where genetic and environmental factors are uniform. A classic example is the intranasal challenge of mice with vesicular stomatitis virus (VSV), which leads to a dichotomous outcome of either survival or death.^9^^,^^10^

The initial phase of a viral infection is governed by the immediate host response to pathogen sensing.^11^^,^^12^ This primary response, which precedes adaptive immunity, is critical as it shapes the entire subsequent disease trajectory. The efficacy of this innate response hinges on the rapid and robust induction of potent antiviral effector programs designed to control viral replication and orchestrate further immune involvement.^13^^,^^14^ Much of the research on these early viral responses has focused on the local site of infection. In contrast, the early systemic response in the peripheral blood remains a key area of investigation, as it is a clinically accessible and ideal source for prognostic biomarkers. Therefore, identifying a predictive signature from blood at the earliest possible time point would be a major advance for timely therapeutic intervention. While some studies have characterized the early systemic immune response, a critical gap remains: few studies have directly linked cellular profiles from a remarkably early time point—such as 24 h post-infection—to the ultimate survival outcome. This represents a major gap in our understanding of how early systemic responses shape the trajectory of a viral infection.

Type I interferons (IFNs) are universally recognized as a cornerstone of the early host defense.^15^ Their rapid induction upon viral sensing triggers a wide range of IFN-stimulated genes (ISGs) that establish an antiviral state in host cells. While the necessity of type I IFN response for viral control is well-established, less is understood about the temporal dynamics of this response. The timing of type I IFN induction and the duration of its signaling can profoundly influence its effects, shifting the balance from a protective, virus-clearing response to a pathological, tissue-damaging one.^16^^,^^17^^,^^18^ However, the degree to which the precise timing of the initial type I IFN surge acts as a critical determinant of an individual’s ultimate outcome remains poorly defined.

Here, we propose that the variability in survival outcomes within genetically uniform mice under highly controlled conditions provides a unique opportunity to uncover the critical, early physiological differences that drive the divergence in infection outcomes, rather than being mere experimental noise. To explore the mechanisms underlying survival versus death following viral infection, we retrospectively analyzed immune cell profiles in the blood immediately before and shortly after infection. This revealed dynamic and transient changes in circulating immune cells that preceded the immune cell infiltration and activation at the infection site, which is previously described.^19^ These early shifts were driven by a transient type I IFN response, which emerged as a key determinant of survival. Importantly, this robust, early type I IFN surge orchestrated a distinct systemic immune landscape, marked by the emergence of a functionally potent, pro-inflammatory neutrophil population characterized by high ICAM1 expression, which was more prominent in the survival group compared to the lethal group. Conditional deletion of ICAM1 in CXCR2^+^ cells resulted in reduced survival following viral infection, highlighting a critical role for ICAM1 in these cells in host protection. Together, these findings establish a new framework in which the precise temporal dynamics of innate immunity, independent of host genetic variation, plays a critical role in shaping the outcome of lethal viral infection.

## Results

### Transient systemic immune dynamics in the peripheral blood during the early phase of VSV infection

**Figure 1:**
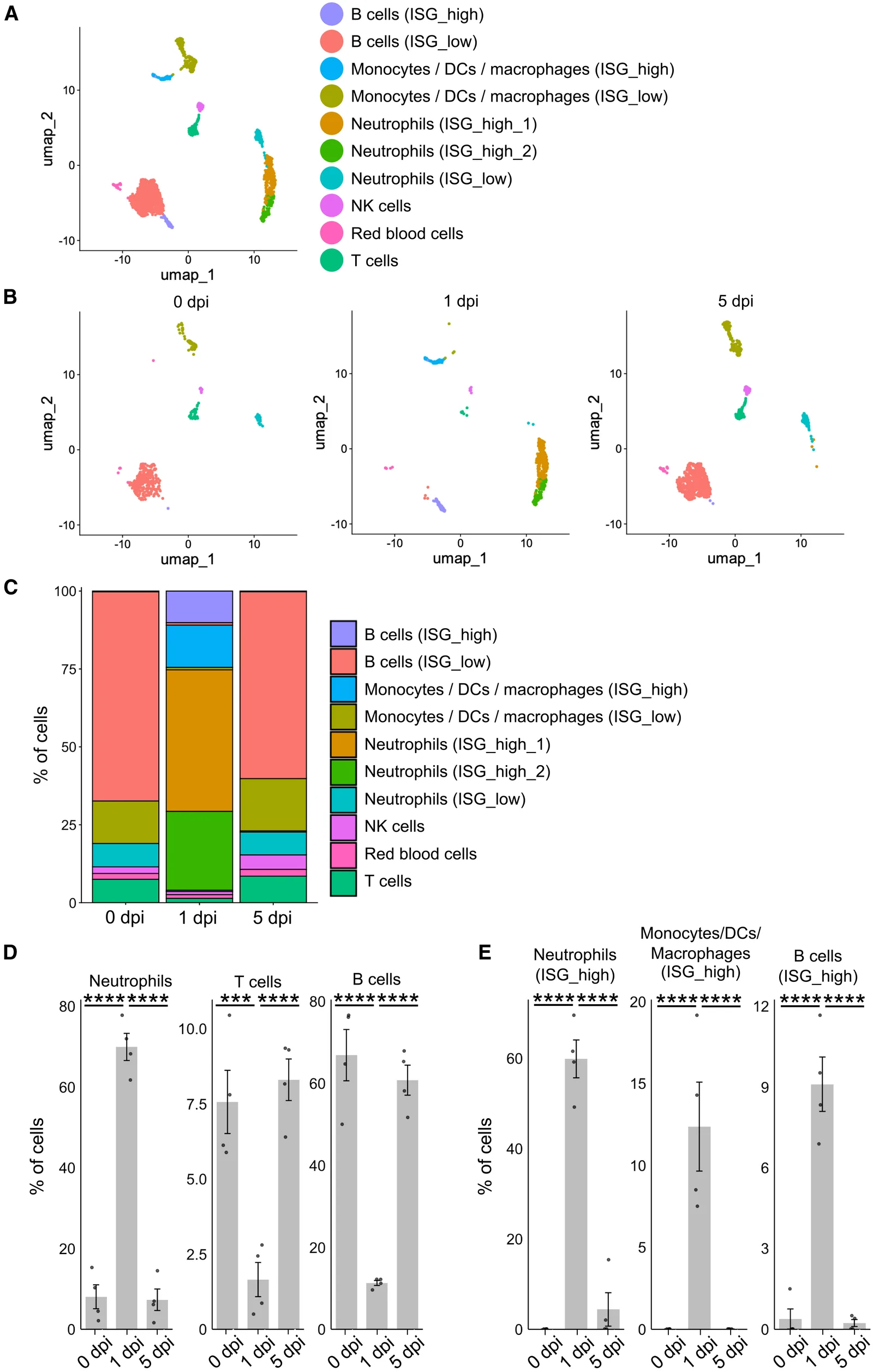
Transient systemic immune remodeling in peripheral blood during early VSV infection (A) Integrated uniform manifold approximation and projection (UMAP) projection of circulating immune cells profiled by single-cell RNA sequencing (scRNA-seq) from the survival and lethal groups in male mice (*n* = 2 each), combining all samples collected at 0, 1, and 5 days post-infection (dpi), shown to provide a global reference for cluster relationships across conditions and time points. (B) UMAP projections from (A) split by time point to show cells at 0, 1, or 5 dpi individually (cell numbers = 374, 502, and 834 cells, respectively). (C) Percentages of each cell cluster within total circulating cells at each time point. (D) Percentages of neutrophils (neutrophils [ISG_high_1] + neutrophils [ISG_high_2] + neutrophils [ISG_low]), B cells (B cells [ISG_high] + B cells [ISG_low]), and T cell clusters within total circulating cells at each time point. *n* = 4 each. (E) Percentages of neutrophils (ISG_high) (neutrophils [ISG_high_1] + neutrophils [ISG_high_2]), monocytes/DCs/macrophages (ISG_high), and B cells (ISG_high) clusters within total circulating cells at each time point. *n* = 4 each. Data in (D) and (E) are shown as mean ± SEM, where each point represents an individual mouse. ∗∗∗*p* < 0.005, ∗∗∗∗*p* < 0.001. Statistical significance was determined using one-way ANOVA with Tukey’s post hoc test (D and E).

While the intranasal VSV infection model is known to induce progressive infiltration of immune cells into the brain beginning around 3 days post-infection (dpi),^19^ the systemic immune dynamics in the peripheral blood remain largely uncharacterized. To investigate early changes in circulating immune cells, we comprehensively mapped the systemic immune landscape by performing single-cell RNA sequencing (scRNA-seq) on peripheral blood from mice at 0, 1, and 5 days post-intranasal VSV challenge (Figures 1A and S1A). This analysis revealed a transient wave of neutrophilia and lymphopenia at 1 dpi, characterized by a marked increase in circulating neutrophils and a parallel decrease in lymphocytes, including B and T cells (Figures 1B–1D), confirmed by flow cytometry analysis (Figure S1B). By 5 dpi, these changes had largely resolved, returning to baseline levels (Figures 1B–1D). Furthermore, a distinct population of ISG-expressing cells (ISG_high clusters) emerged in the blood at 1 dpi and disappeared by 5 dpi (Figure 1E). Together, these findings suggest that intranasal VSV infection triggers a rapid and transient remodeling of circulating immune cells, coupled with a systemic antiviral response during the early phase of infection.

### Transient type I IFN production contributes to systemic immune remodeling

**Figure 2:**
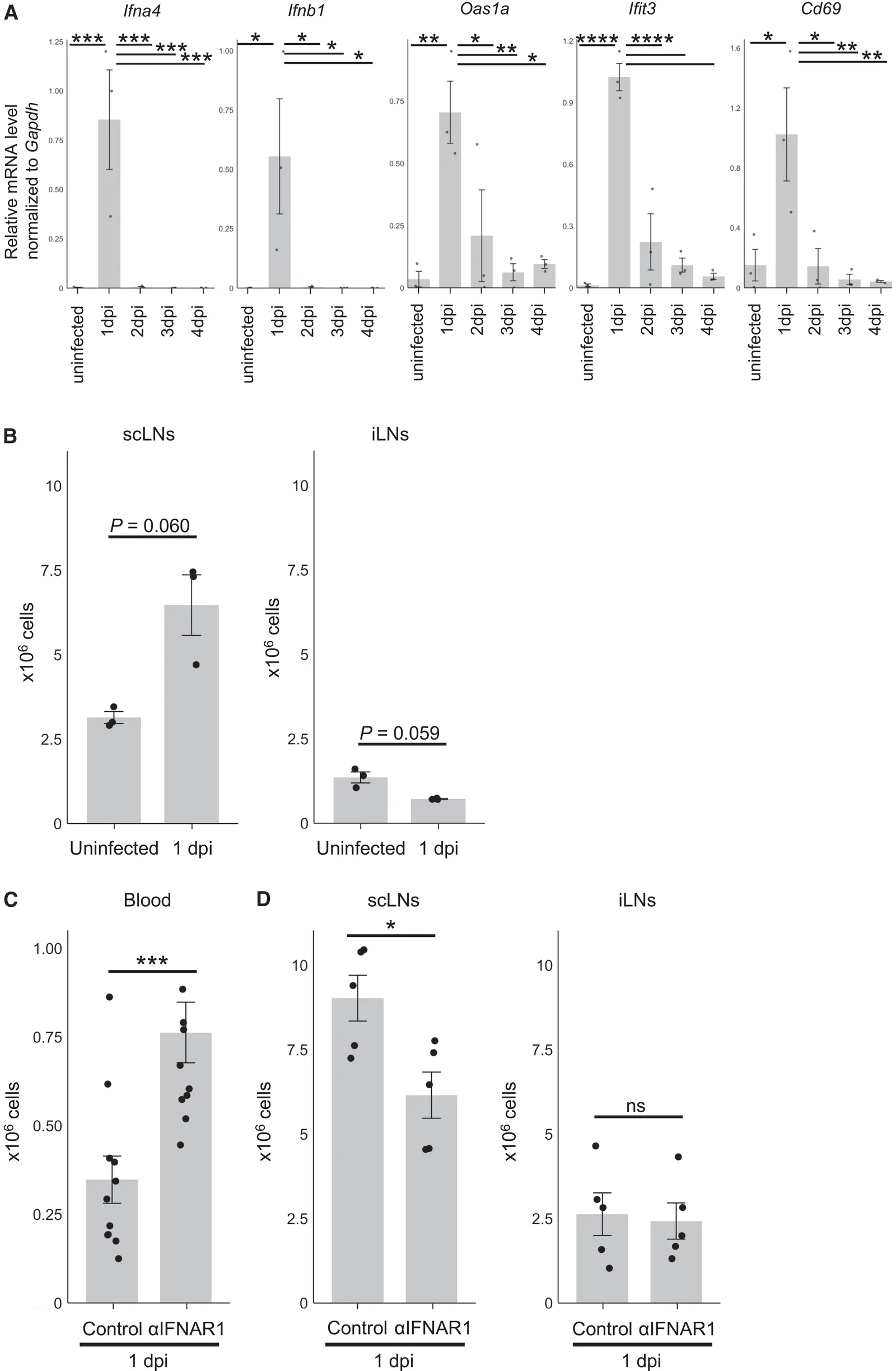
Transient type I interferon induction in draining lymph nodes mediates lymphocyte retention and systemic immune remodeling (A) Relative mRNA levels of *Ifna4*, *Ifnb1*, *Oas1a*, *Ifit3*, and Cd69 measured by RT-qPCR in superficial cervical lymph nodes (scLNs) from uninfected and infected male mice at the indicated dpi (*n* = 3 each). (B) Flow cytometric analysis of absolute numbers of CD45^+^CD11b^−^ lymphocytes in the scLNs and inguinal lymph nodes (iLNs) from uninfected and infected male mice at 1 dpi (*n* = 3 each). (C) Flow cytometric analysis of absolute numbers of circulating CD45^+^CD11b^−^ lymphocytes at 1 dpi from male mice treated with anti-IFNAR1 antibody or isotype IgG at 0 dpi (*n* = 11 each). (D) Flow cytometric analysis of absolute numbers of CD45^+^CD11b^−^ lymphocytes in the scLNs and iLNs at 1 dpi from male mice treated with anti-IFNAR1 antibody or isotype IgG at 0 dpi (*n* = 6 each). Data are presented as mean ± SEM (A–C). ns, not significant; ∗*p* < 0.05, ∗∗*p* < 0.01, ∗∗∗*p* < 0.005, ∗∗∗∗*p* < 0.001. Statistical significance was determined using one-way ANOVA with Tukey’s post hoc test (A); an unpaired, two-tailed Welch’s *t* test (B and D); or an unpaired, two-tailed Mann-Whitney *U* test (C).

Since the ISG-high population transiently emerged in the circulation, we hypothesized that the dynamic changes in circulating immune cells at 1 dpi are driven by an early wave of type I IFN production. VSV infection is known to induce type I IFN not only at the site of infection but also in the draining nodes,^20^ lung,^21^ and spleen,^22^ thereby influencing systemic immune cell states.^23^ We therefore examined type I IFN induction in the superficial cervical lymph nodes (scLNs), which drain the nasal lymphatics and cerebrospinal fluid (CSF)^24^ and thus serve as the primary draining nodes in this infection model, as well as in the lung and spleen. Daily analysis of scLNs from 0 to 4 dpi revealed a sharp and transient induction of IFN-α and IFN-β expression specifically at 1 dpi, accompanied by upregulation of multiple ISGs (Figures 2A, S2A, and S2B). Analysis of lung and spleen revealed a similar transient increase in IFN expression at 1 dpi, although the magnitude of induction relative to baseline was less pronounced than in the scLNs (Figures S3A and S3B). Together, these results indicate that type I IFN responses occur systemically, with particularly prominent and well-defined transient induction in the scLNs. Because type I IFNs promote lymphocyte retention within lymphoid organs,^25^ this transient IFN surge may underlie the observed reduction in circulating lymphocytes at 1 dpi by promoting their retention in the scLN. Indeed, the expression of CD69—a key regulator of lymphocyte retention in lymphoid organs by type I IFNs^25^—was also transiently elevated at 1 dpi (Figure 2A). Consistently, accumulation of lymphocytes was observed in the scLNs but not in non-draining inguinal lymph nodes (iLNs) at 1 dpi (Figure 2B), when circulating lymphocytes drastically decreased (Figure S1B), suggesting that the reduction of circulating T and B cells at 1 dpi likely reflects their retention in draining lymph nodes, which could facilitate efficient interactions between antigen-presenting cells and antigen-specific lymphocytes during the early phase.^26^^,^^27^^,^^28^ Moreover, the transient nature of this lymphocyte reduction is consistent with the short-lived type I IFN response, which peaked at 1 dpi and subsided thereafter (Figures 1E and 2A). Notably, type I IFN signaling blockade by the administration of an anti-IFNAR1 antibody at 0 dpi attenuated the transient lymphopenia, resulting in significantly higher numbers of circulating lymphocytes at 1 dpi compared to control (Figure 2C). Consistent with these results, IFNAR1 blockade also reduced the accumulation of lymphocytes in the scLNs without affecting lymphocyte numbers in the iLNs (Figure 2D), suggesting that recruitment of circulating lymphocytes to the draining lymph node occurs in a type I IFN-dependent manner. Collectively, these data indicate that a short-lived burst of type I IFN production orchestrates the rapid and coordinated remodeling of circulating immune cells and the activation of systemic antiviral programs.

### Temporal requirement of type I IFN signaling governs a critical window of survival following VSV infection

**Figure 3:**
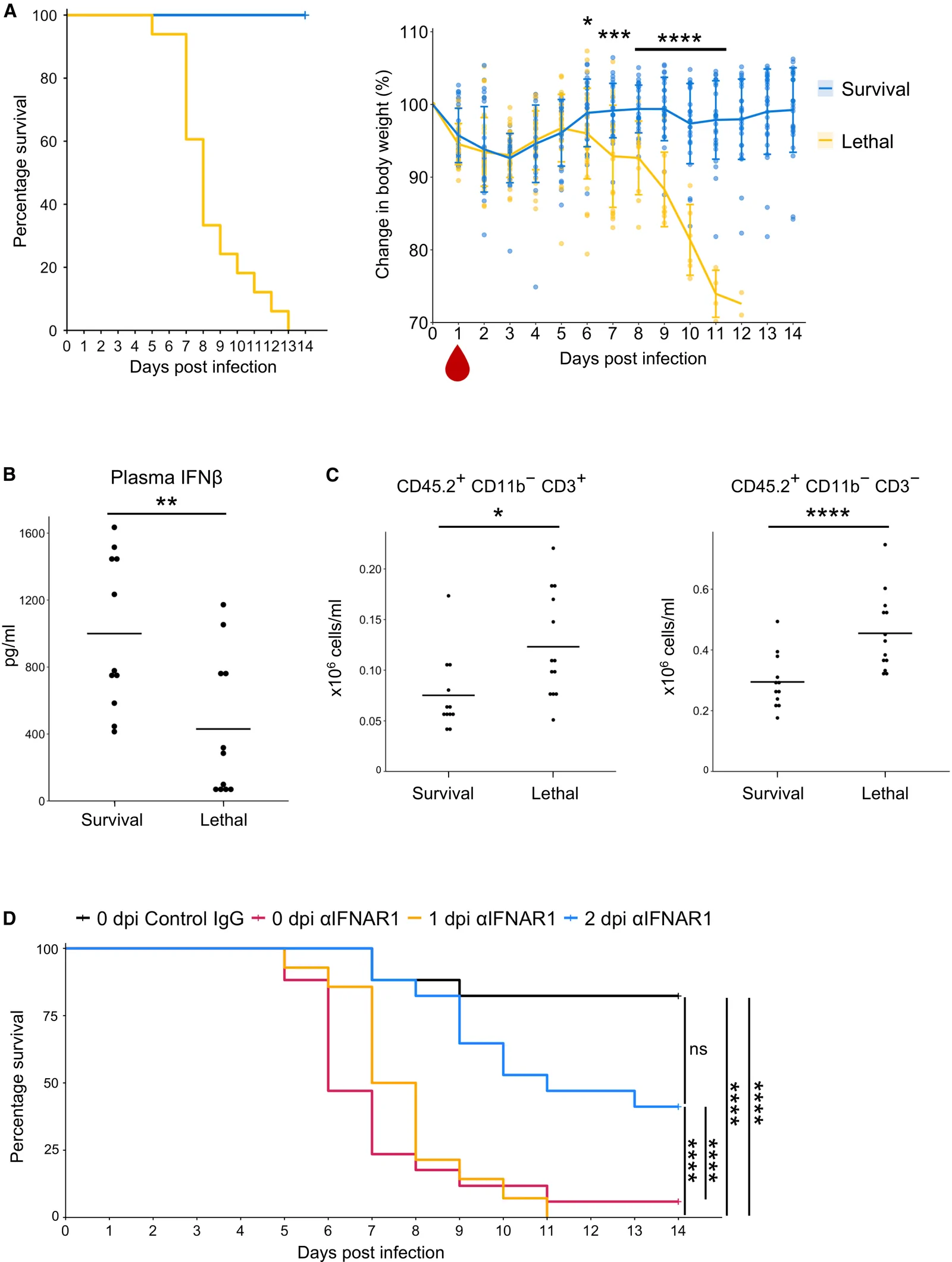
A temporally restricted type I IFN surge is essential for the survival (A) Survival curves and body weight changes of male mice classified as the survival group (survived ≥14 dpi) or the lethal group (died or reached the humane endpoint defined as body weight <70% of the initial weight before 14 dpi) (*n* = 23 and 33, respectively). (B) IFNβ levels in the plasma from male mice in the survival and lethal groups (*n* = 11 each) at 1 dpi, measured by ELISA. (C) Flow cytometric analysis of absolute numbers of circulating CD45^+^ CD11b^−^ CD3^+^ and CD45^+^ CD11b^−^ CD3^−^ lymphoid cells from male mice in the survival and lethal groups at 1 dpi (*n* = 12 and 13, respectively). (D) Survival curves of male mice treated with anti-IFNAR1 at 0 dpi (*n* = 17), 1 dpi (*n* = 14), or 2 dpi (*n* = 17), or with isotype IgG at 0 dpi (*n* = 17). Data in (A) are presented as mean ± SD. Data in (B) and (C) are shown as a dot plot where each point represents an individual mouse and the horizontal line indicates the mean. ∗∗*p* < 0.01. Statistical significance was determined using an unpaired, two-tailed Mann-Whitney *U* test (A–C) or the Wilcoxon (Breslow) test with Benjamini-Hochberg (BH) correction for multiple comparisons (D).

Given that type I IFN is essential for antiviral defense, we hypothesized that divergent survival outcomes after intranasal VSV challenge might stem from differences in the initial type I IFN response. To test this, mice were retrospectively classified into survival and lethal groups (Figure 3A), and plasma IFN-β levels were measured at 1 dpi (Figure 3B). Strikingly, IFN-β levels were significantly higher in the survival group than in the lethal group, indicating that a robust early type I IFN response is associated with a favorable outcome. This notion is corroborated by the observation that the survival group, which had higher IFN-β levels, similarly exhibited a more pronounced reduction in circulating lymphocytes at 1 dpi (Figure 3C). To directly evaluate the functional requirement for type I IFN signaling, we administered an anti-IFNAR1 antibody. Blocking type I IFN signaling markedly reduced survival when treatment was initiated at 0 dpi or 1 dpi but, importantly, had a less pronounced effect when given at 2 dpi (Figure 3D). These findings identify a narrow and critical window immediately after infection during which type I IFN signaling is indispensable, demonstrating that the timing of this response is a key determinant of disease outcome.

### ICAM1 expression defines an early neutrophil signature associated with survival outcome

**Figure 4:**
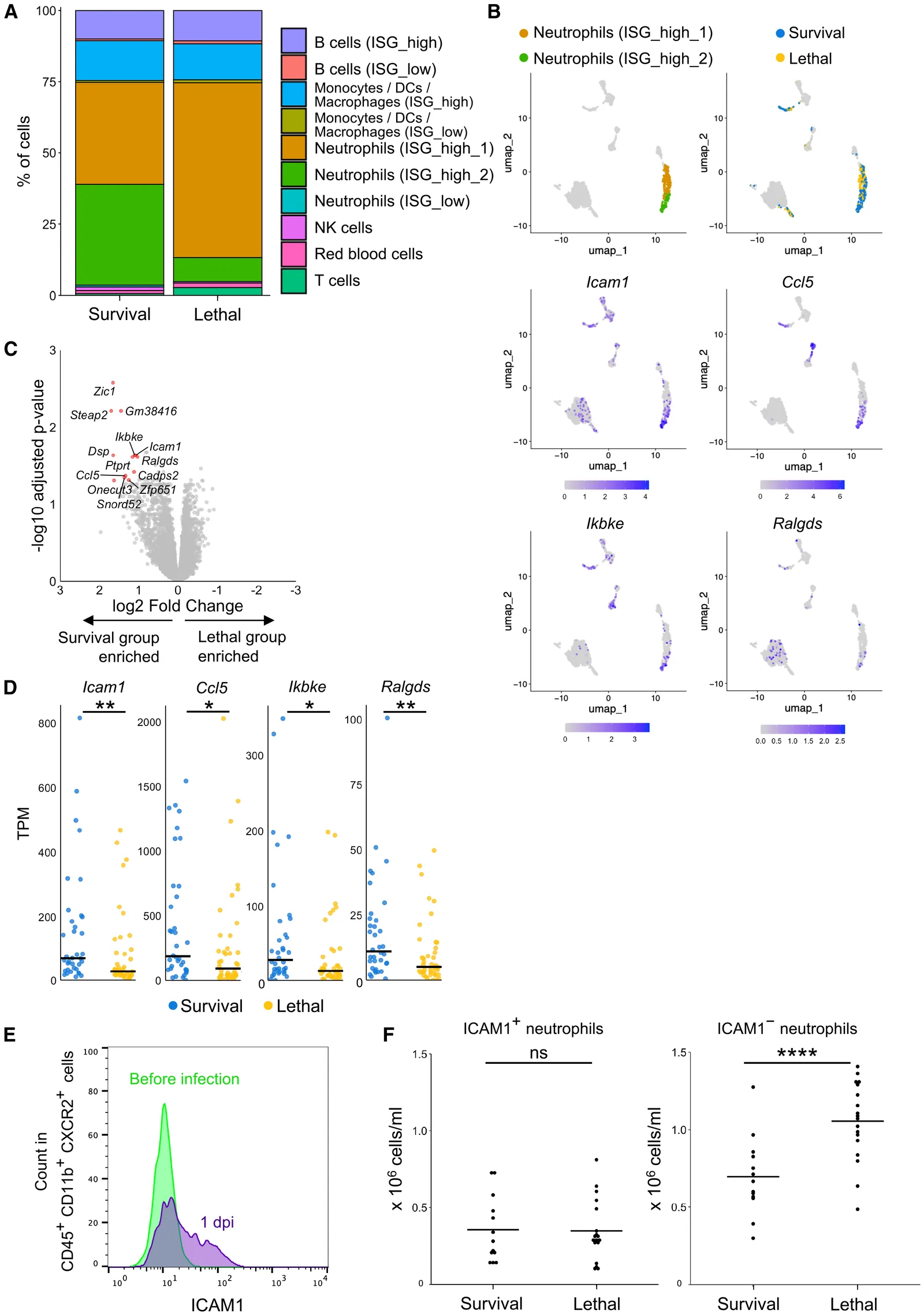
ICAM1 expression distinguishes early neutrophil heterogeneity associated with survival outcome (A) Percentages of each scRNA-seq-defined cell cluster within total circulating cells in the survival and lethal groups at 1 dpi. (B) UMAP and feature plots from scRNA-seq analysis, showing expression of key differentially expressed genes (DEGs) between neutrophils (ISG_high_1) and neutrophils (ISG_high_2). (C) Volcano plot depicting DEGs identified by bulk RNA-seq of sorted circulating CXCR2^+^ neutrophils from male mice in the survival group versus the lethal group (*n* = 36 and 42, respectively) at 1 dpi. Genes with an absolute log_2_ fold change >1 and an adjusted *p* value < 0.05 are highlighted in red. (D) Expression levels (TPM; transcripts per million) of key DEGs identified in both the bulk RNA-seq of sorted circulating CXCR2^+^ neutrophils and the scRNA-seq of neutrophils. (E) Representative flow cytometry histograms of ICAM1 surface expression on blood neutrophils (CD45^+^CD11b^+^CXCR2^+^) before infection and at 1 dpi. (F) Flow cytometric analysis of absolute numbers of ICAM1^+^ and ICAM1^−^ neutrophils in the blood of male mice in the survival and lethal groups (*n* = 13 and 18, respectively) at 1 dpi. Data in (D) and (F) are shown as a dot plot where each point represents an individual mouse and the horizontal line indicates the mean. ns, not significant; ∗*p* < 0.05, ∗∗*p* < 0.01, ∗∗∗∗*p* < 0.001. Statistical significance was determined using an unpaired, two-tailed Mann-Whitney *U* test in (D) and (F).

Given that an early type I IFN surge is associated with survival, we next compared the systemic immune profiles of the survival and lethal groups to characterize the cellular response during this critical window. The most striking difference was observed in the neutrophil compartment. Specifically, a neutrophil cluster designated ISG_high_2 constituted a higher percentage of circulating cells in the survival group, while the ISG_high_1 cluster was more prevalent in the lethal group (Figure 4A).

We next sought to further characterize the neutrophil subsets that correlate with survival. We compared the transcriptional differences between the survival-associated ISG_high_2 neutrophil cluster and the lethal-associated ISG_high_1 neutrophil cluster, which revealed that genes related to innate immune responses and pro-inflammatory signaling pathways were enriched in the survival-associated ISG_high_2 neutrophil cluster (Figures S4A and S4B), such as *Icam1* and *Ccl5* (Figure 4B). We also performed bulk RNA-seq on sorted CXCR2^+^ neutrophils, which corroborated the scRNA-seq findings, demonstrating that neutrophils from the survival group expressed significantly higher levels of these signature genes (Figures 4C and 4D). Among these, we focused on Icam1 because it showed robust upregulation in the survival group. In addition, its cell surface expression allows detection by flow cytometry, making it a strong candidate for a prognostic predictor. Hence, we examined ICAM1 protein levels on neutrophils by flow cytometry. Surface ICAM1 expression was upregulated at 1 dpi compared to baseline (Figures 4E and S4C). Importantly, the lethal group exhibited a significantly higher abundance of ICAM1^−^ neutrophils at 1 dpi (Figure 4F). This suggests that the proportional differences in neutrophil clusters observed by scRNA-seq are primarily driven by the predominance of the ICAM1^−^ population in the lethal group. Collectively, these multi-platform analyses pinpoint the early polarization of circulating neutrophils, distinguished by differential ICAM1 expression, as a primary cellular correlate of survival.

### Systemic type I IFN acts on the bone marrow to induce ICAM1 expression on neutrophils

To establish a mechanistic link between the early type I IFN surge and the subsequent emergence of ICAM1^+^ neutrophils, we investigated whether type I IFN could directly regulate ICAM1 expression. Neutrophils are generated from myeloid progenitors in the bone marrow (BM) and subsequently enter the circulation.^29^ Thus, type I IFN could influence ICAM1 expression on neutrophils both in the BM and in the circulation. To test these possibilities, we administered IFN-β to mice and analyzed neutrophils in both peripheral blood and BM 24 h later. IFN-β treatment did not increase the number of ICAM1^+^ neutrophils in peripheral blood; however, it significantly increased the number of ICAM1^+^ neutrophils in the BM in a concentration-dependent manner (Figures S5A–S5C). These findings led us to hypothesize that infection-induced type I IFN responses act within the BM to generate an ICAM1^+^ neutrophil population, which later appears as distinct subsets in the circulation. To test the prediction that the survival group should exhibit a stronger BM type I IFN response, we retrospectively compared transcriptomic profiles of BM cells. Specifically, we performed BM biopsy at 1 dpi, which represents an early time point for the divergence of disease outcomes. We then classified the samples into survival and lethal groups based on their ultimate fate. This analysis revealed that genes expressed higher in the survival group were significantly enriched for pathways associated with IFN-β responses (Figures S6A and S6B). Together, these results support a model in which mice in the survival group mount a more robust type I IFN response in the BM, leading to an increased number of ICAM1^+^ neutrophils. This early priming at the BM may underlie the differences in circulating neutrophil subsets associated with survival outcomes.

### ICAM1 defines a transcriptionally and functionally distinct neutrophil subset required for host protection

**Figure 5:**
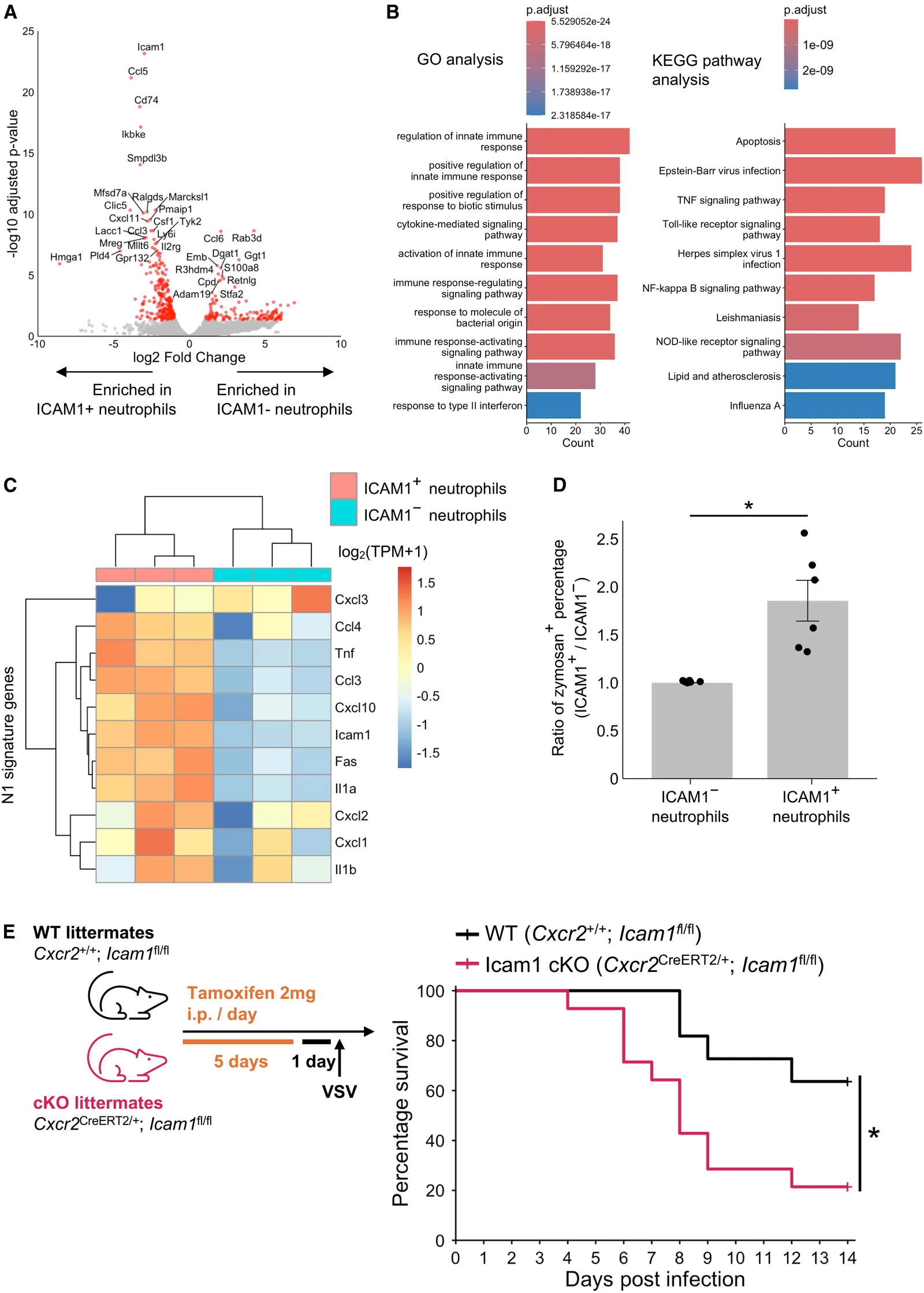
ICAM1 expression distinguishes transcriptionally and functionally distinct neutrophil subsets associated with survival outcome (A) Volcano plot depicting DEGs identified by bulk RNA-seq in ICAM1^+^ neutrophils compared to ICAM1^−^ neutrophils from sorted blood samples of male mice (*n* = 3 per group) at 1 dpi. Genes with an absolute log_2_ fold change > 1 and adjusted *p* value < 0.05 are highlighted in red. (B) Gene Ontology (GO) and Kyoto Encyclopedia of Genes and Genomes (KEGG) pathway analysis of 293 genes more highly expressed in ICAM1^+^ neutrophils compared to ICAM1^−^ neutrophils (adjusted *p* value < 0.05, log_2_ fold change < −1). *p* values were determined by a hypergeometric test with BH correction. (C) Heatmap showing the expression of N1 signature genes in sorted ICAM1^+^ and ICAM1^−^ neutrophils. (D) Flow cytometric analysis of *in vivo* phagocytic activity of ICAM1^+^ versus ICAM1^−^ neutrophils from male mice, measured as the percentage of cells that engulfed fluorescently labeled zymosan particles. For each mouse, the value for the ICAM1^+^ population was normalized to that of the ICAM1^−^ counterpart (set as 1) (*n* = 6). (E) Schematic representation and survival curves of age- and sex-matched *Cxcr2*^+/+^; *Icam1*^fl/fl^ (wild type [WT], *n* = 11) and *Cxcr2*^CreERT2/+^; *Icam1*^fl/fl^ (Icam1 cKO, *n* = 14) littermates. Data in (D) are presented as mean ± SEM. ∗*p* < 0.05. Statistical significance was determined using a one-sample *t* test (D) or the Wilcoxon (Breslow) test with Benjamini-Hochberg (BH) correction for multiple comparisons (E).

To gain insights into whether these two neutrophil populations exhibit distinct antiviral properties, we first compared the transcriptomic differences between two neutrophil populations. We performed RNA-seq on sorted ICAM1^+^ and ICAM1^−^ neutrophils, which revealed that ICAM1^+^ neutrophils were enriched for genes related to innate immune responses and pro-inflammatory signaling pathways (Figures 5A and 5B). This transcriptomic profile mirrors that of the survival-associated ISG_high_2 cluster compared to the lethal-associated ISG_high_1 cluster in scRNA-seq analysis (Figures S4A and S4B). In contrast, genes highly expressed in ICAM1^−^ neutrophils did not reveal clear enrichment in any specific pathways (Figure S7A). The highly inflammatory transcriptional signature of ICAM1^+^ neutrophils is reminiscent of the N1 polarization of neutrophils in tumor microenvironment.^30^ Indeed, ICAM1^+^ neutrophils were significantly enriched for a pro-inflammatory N1-like signature compared to ICAM1^−^ neutrophils (Figure 5C), which is consistent with higher expression of N1 signature genes in the survival-associated ISG_high_2 cluster relative to the lethal-associated ISG_high_1 cluster (Figure S7B). These results suggest that survival-associated neutrophils resemble the transcriptome of functionally competent N1 phenotype described in tumor microenvironments, in the context of acute viral infection.

Next, we asked whether the transcriptional polarization we observed is coupled with functional divergence in our model. ICAM1^+^ neutrophils have been reported to be a potent cell population, exhibiting enhanced effector functions such as greater phagocytic activity in a murine model of endotoxemia.^31^ To determine if this functional advantage holds true in the context of viral infection, we assessed their phagocytic function. Indeed, circulating ICAM1^+^ neutrophils from VSV-infected mice at 1dpi showed significantly higher phagocytic activity than their ICAM1^−^ counterparts (Figure 5D). This indicates that ICAM1^+^ neutrophils represent a functionally more potent subset during the viral infection. Collectively, these findings reveal that ICAM1 expression defines a transcriptional and functional polarization of neutrophils, distinguishing a potent, pro-inflammatory subset from a distinct, less-activated population.

To directly evaluate the functional role of ICAM1 on neutrophils, we generated neutrophil-specific ICAM1-deficient mice (Icam1fl/fl Cxcr2-CreERT2). Strikingly, the loss of ICAM1 in neutrophils significantly reduced survival following VSV infection (Figure 5E), demonstrating that ICAM1 expression on neutrophils is a functional requirement for host protection.

## Discussion

A fundamental challenge in infectious disease research is elucidating the precise early events that determine the ultimate clinical outcome. This is particularly difficult because most studies, especially in humans, rely on samples collected well after the initial infection or the onset of symptoms. By capturing the immune profile during the initial phase of infection and retrospectively correlating it with survival, our study not only provides a detailed characterization of systemic immune responses—such as type I IFN-mediated lymphocyte migration and myeloid activation—at an exceptionally early stage (24 h post-infection) but also directly links these events to the host’s ultimate fate. This early divergence in the peripheral circulation is particularly striking, as it indicates that a prognostic systemic immune signature emerges prior to the development of major cellular changes at the olfactory bulb, the primary site of intranasal VSV infection.^19^ Thus, while the critical host-pathogen interactions ultimately unfold within the infection site, our findings suggest that the trajectory toward survival or death is reflected in—and likely shaped by—systemic immune events established far earlier than previously appreciated.

An intuitive explanation for such variability could be minor differences in the initial infectious dose. However, our data do not support this, as the survival rate was largely independent of the viral load within the tested range (Figure S8A), indicating that viral dose is not the primary determinant in our model. Similarly, we considered pre-existing host-intrinsic factors known to influence infection outcomes, such as basal type I IFN levels^32^ or stress hormones^33^^,^^34^; yet, we found no significant pre-infection differences in these variables between the prospective survival and lethal groups (Figures S8B–S8D). The exclusion of these potential confounders suggests that the divergence in survival is governed by previously unappreciated variability that cannot be explained by viral load or basal physiological states, thereby positioning our model as a powerful platform to identify previously unrecognized regulators of host response heterogeneity.

A central finding of this study is the identification of ICAM1 expression on neutrophils as both a predictive and functional determinant of disease outcome. Although ICAM1 upregulation on neutrophils is detectable in peripheral blood and has been reported under inflammatory conditions in humans,^35^^,^^36^^,^^37^ its direct relationship to disease severity and its precise functional significance have remained unclear. Here, we demonstrate that ICAM1 expression in CXCR2^+^ cells is indispensable for host survival following viral infection, establishing ICAM1 not merely as a marker of inflammation but as a functional component of host protection in viral infection. This specialized, protective role challenges the conventional paradigm of neutrophil involvement in viral pathogenesis, where these cells are often perceived as secondary or even detrimental. Importantly, the ICAM1^+^ subset is distinct from other recently described functional neutrophil subpopulations; for instance, while IFN-induced Ly6E^+^ neutrophils enhance T cell activity in tumors,^38^ and OLFM4^+^ neutrophils play detrimental roles in bacterial infections,^39^ we found that *Ly6e* and *Olfm4* expression did not correlate with the ICAM1^+^-mediated protection in our model (Figures S9A–S9C). These findings establish the ICAM1^+^ neutrophil as a transcriptionally and functionally unique subpopulation that is essential for shaping host survival during the early phase of viral infection.

The identification of this survival-associated state underscores the critical importance of innate immune timing. While sustained type I IFN signaling and pro-inflammatory neutrophils are often associated with immunopathology and severe outcomes in the later stages of viral infections,^3^^,^^5^^,^^40^^,^^41^^,^^42^^,^^43^ our data suggest that the beneficial role of the type I IFN-neutrophil axis is strictly restricted to the early phase. We further propose that this early protective response is rooted in the BM, where type I IFN modulates neutrophil heterogeneity during their development. Given that elevated ICAM1 expression is associated with stress myelopoiesis,^44^^,^^45^ the ICAM1^+^ population may represent a cohort of newly generated cells emerging from enhanced myelopoiesis triggered by the initial antiviral response. Validating these immune signatures as both prognostic biomarkers and functional determinants could open new avenues for host-directed therapies aimed at promoting protective immune responses.

The mechanisms by which this neutrophil heterogeneity dictates the survival outcome remain an important area for future investigation. Our findings, including the reduced survival observed upon conditional deletion of ICAM1 in CXCR2^+^ cells, establish ICAM1 as a functional component of this process. One possibility is that the ICAM1^+^ population directly confers protection. ICAM1 expression on neutrophils is associated with enhanced anti-pathogenic activities,^31^^,^^46^ including phagocytosis, suggesting these cells have a greater capacity to eliminate the virus. Furthermore, as a canonical adhesion molecule, ICAM1 plays a fundamental role in mediating cell-cell interactions. Since ICAM1^+^ neutrophils have been reported to modulate lymphocyte activity via direct contact,^47^^,^^48^ this population may further contribute to survival by shaping an optimal adaptive immune response. Conversely, ICAM1 deletion may have favored a detrimental outcome by shifting the neutrophil population toward an ICAM1^−^ state. While ICAM1^−^ neutrophils do not exhibit specific pathway enrichment or fully recapitulate a canonical immunosuppressive N2 phenotype at the transcriptomic level in our dataset (Figures S10A and S10B), their predominance in the lethal group—which exhibits a weaker type I IFN response—raises the possibility that they are not simply less-activated bystanders. Rather, this population, emerging in the context of a suboptimal type I IFN response, may actively contribute to a detrimental outcome, perhaps through ineffective viral control or other uncharacterized pathogenic functions. Therefore, future studies should investigate both the protective functions of ICAM1^+^ neutrophils and the potentially harmful roles of their ICAM1^−^ counterparts.

Our study also presents a methodological advance in overcoming the logistical and technical hurdles of retrospective transcriptomic analysis in high-containment viral models. A major constraint in retrospective designs is that samples must be archived until the final prognosis is settled, precluding the immediate processing of fresh cells. To overcome this challenge, we combined a cell stabilization reagent with a cryopreservation solution, enabling the stable long-term storage of samples (see STAR Methods). This approach provided a critical logistical benefit, allowing us to select and analyze samples retrospectively based on the definitive survival outcome. Furthermore, for RNA-seq analysis following fluorescence-activated cell sorting (FACS), viral inactivation via fixation is often mandatory but typically compromises RNA extraction efficiency. We applied a thio-bis succinimidyl propionate fixation method,^49^ which we confirmed successfully inactivated the virus while effectively preserving the integrity of labile RNA. These optimized protocols were also instrumental in combating a significant technical challenge in the transcriptomic profiling of circulating immune cells: the instability of neutrophils. Neutrophils are highly susceptible to *ex vivo* apoptosis and contain a low amount of RNA and abundant intracellular RNases, making their RNA notoriously difficult to analyze.^50^ Consequently, many transcriptomic studies of blood focus on the more stable peripheral blood mononuclear cell (PBMC) fraction, often overlooking the neutrophil compartment. Our methodology was therefore essential for robustly profiling these fragile populations and ultimately identifying the ICAM1-defined neutrophil subset as a key mediator of survival.

Beyond these potential applications in both clinical and basic research methodology, our work establishes a conceptual framework: treating innate biological variability not as experimental noise but as a discovery tool capable of revealing critical early checkpoints in pathogenesis that are otherwise hidden. This approach opens the door to a new understanding of individuality in disease, where the outcome is shaped not only by the genetic blueprint but also by the emergent dynamics of the initial immune response.

### Limitations of the study

While our study identifies ICAM1^+^ neutrophils as a key determinant of survival during early viral infection, several limitations remain. First, although our data demonstrate a functional requirement for ICAM1 expression on neutrophils, the precise mechanisms by which ICAM1^+^ neutrophils confer protection require further investigation.

Second, our findings are based on a single viral infection model, and it remains unclear to what extent this protective neutrophil state is conserved across other viral infections with distinct tropisms and immunopathological features.

Finally, although ICAM1 upregulation on neutrophils has been reported in humans, the presence, functional relevance, and prognostic value of this ICAM1^+^ neutrophil subset in human viral infections remain to be established. Future studies addressing these points will be important to generalize and translate our findings.

## Resource availability

### Lead contact

Requests for further information and resources should be directed to and will be fulfilled by Tomohiko Okazaki (okazaki@igm.hokudai.ac.jp).

### Materials availability

The genetically manipulated mice are available from the lead contact on reasonable request.

### Data and code availability

scRNA-seq and bulk RNA-seq data have been deposited in the Gene Expression Omnibus (GEO) under accession number GSE313167, which serves as a SuperSeries comprising the following datasets: GSE313019 (scRNA-seq), GSE313732 (RNA-seq of blood-derived CXCR2^+^ cells from survival and lethal groups at 1 dpi), GSE313164 (RNA-seq of bone marrow-derived CXCR2^+^ cells from survival and lethal groups at 1 dpi), GSE313165 (RNA-seq of blood-derived ICAM1^+^ CXCR2^+^ and ICAM1^−^ CXCR2^+^ cells at 1 dpi), and GSE325974 (RNA-seq of blood-derived CXCR2^+^ cells from survival and lethal groups pre-infection). All other data are available from the corresponding author upon reasonable request.

## Acknowledgments

We thank all members of the Laboratory of Molecular Biology (Graduate School of Pharmaceutical Sciences, The University of Tokyo) and the Laboratory of Molecular Cell Biology (Institute for Genetic Medicine, Hokkaido University) community for feedback throughout this project. We also thank Maki Ukai-Tadenuma, Akira Kamei, and Emiko Kobayashi (ES-Mouse/Virus Core, The University of Tokyo) for generating Icam1-flox mice line. R.S. was supported by the 10.13039/501100001700Ministry of Education, Culture, Sports, Science, and Technology of Japan (MEXT) JP23KJ0674. A.S. was supported by 10.13039/501100001700MEXT JP22KJ1176. H.S. was supported by the 10.13039/100009619Japan Agency for Medical Research and Development (AMED) JP223fa627001 (UTOPIA Young Researcher Development Program). Y.G. was supported by MEXT JP22H00431, MEXT JP22H04925 (PAGS), MEXT JP24H02322, MEXT JP18gm0610013, and MEXT JP24gm1310004. T.O. was supported by the MEXT JP24K02172, MEXT J24K22007, the FOREST program of the 10.13039/501100002241Japan Science and Technology Agency JPMJFR204Q, JP25gm6910022, Takano Life Science Research Foundation, 10.13039/100024224Chemo-Sero-Therapeutic Research Institute, 10.13039/501100004398Mitsubishi Foundation, 10.13039/100007428Naito Foundation, Secom Foundation, 10.13039/501100007263Astellas Foundation for Research on Metabolic Disorders, Takeda Science Foundation, and Suntory Rising Stars Encouragement Program in Life Sciences.

## Author contributions

Conceptualization, R.S. and T.O.; methodology, R.S., H.S., and H.U.; software, R.S. and H.S.; formal analysis, R.S. and H.S.; investigation, R.S., Y.D., K.S., F.Y., H.U., and A.S.; writing – original draft, R.S. and T.O.; writing – review and editing, all authors; supervision, Y.G. and T.O.; project administration, T.O.; funding acquisition, H.S., Y.G., and T.O.

## Declaration of interests

R.S. and A.S. are employees of Daiichi Sankyo Co., Ltd. R.S. and A.S. were students at The University of Tokyo at the time of this study. Daiichi Sankyo Co., Ltd., was not involved in this study.

## Declaration of generative AI and AI-assisted technologies in the writing process

During the preparation of this work, the authors used ChatGPT to assist with language editing and improvement of manuscript text. After using this tool, the authors reviewed and edited the content as needed and take full responsibility for the content of the publication.

## STAR★Methods

### Key resources table

**Table:** 

REAGENT or RESOURCE	SOURCE	IDENTIFIER
**Antibodies**		

PerCP/Cyanine5.5 anti-mouse CD45.2 clone 104	Biolegend	Cat# 109828; RRID: AB_893350
APC/Cyanine7 anti-mouse CD11b clone M1/70	Biolegend	Cat# 101226; RRID: AB_830642
Purified anti-mouse CD16/32 clone 93	Biolegend	Cat# 101302; RRID: AB_312801
PE anti-mouse CD182(CXCR2) clone SA044G4	Biolegend	Cat# 149304; RRID: AB_2565692
APC anti-mouse CD54(ICAM1) clone YN1/1.7.4	Biolegend	Cat# 116129; RRID: AB_2800582
FITC anti-mouse CD3 Antibody clone 17A2	Biolegend	Cat# 100204; RRID: AB_312661
MicroBeads anti-PE	Miltenyi Biotec	Cat# 130-048-801; RRID: AB_244373
Mouse IgG1 isotype control-InVivo	Selleck	Cat# A2106; RRID: AB_3722689
Anti-mouse IFNAR-1-InVivo	Selleck	Cat# A2121; RRID: AB_2927720
InVivoMAb rat IgG2b isotype control clone LFT-2	Bio X Cell	Cat# BE0090; RRID: AB_1107780
InVivoMAb anti-mouse CD317 clone 927	Bio X Cell	Cat# BE0311; RRID: AB_2736991
BD Mouse Immune Single-Cell Multiplexing Kit	BD	Cat# 633793; RRID: AB_2870301

**Bacterial and virus strains**		

Vesicular stomatitis virus (VSV) serotype New Jersey	Tadatsugu Taniguchi Lab at the University of Tokyo	N/A

**Chemicals, peptides, and recombinant proteins**		

Recombinant Mouse IFN-β1 (carrier-free)	Biolegend	581302
Zymosan A BioParticles, Alexa Fluor 488 conjugate	Invitrogen	Z23373
Clodronate liposome and control reagent	HYG	16003641
Albumin, from Bovine Serum (BSA), Fatty Acid Free	Fujifilm	011–15144
RNAiso Plus	Takara	9109
Dithio-bis succinimidyl propionate (DSP)	Thermo Scientfic	22586
Paraformaldehyde	Sigma-Aldrich	441244
10%-Formaldehyde Neutral Buffer Solution pH6.9–7.1	Nacalai	37152–51
Crystal Violet	Nacalai	09803–62
Methyl Cellulose #4000	Nacalai	22224–42

**Deposited data**		

Mouse blood scRNA-seq	This paper	GSE313019
Mouse blood CXCR2^+^ neutrophil RNA-seq at 1dpi	This paper	GSE313732
Mouse peripheral bone marrow cell RNA-seq	This paper	GSE313164
Mouse ICAM1^+^ and ICAM1^−^ neutrophil RNA-seq	This paper	GSE313165
Mouse blood CXCR2^+^ neutrophil RNA-seq pre-infection	This paper	GSE325974

**Experimental models: Organisms/strains**		

Mouse: WT C57BL/6JJcl	CLEA Japan	RRID:MGI:3055581

**Oligonucleotides**		

Mouse *Gapdh* F ATGAATACGGCTACAGCAACAGG	ThermoFisher	N/A
Mouse *Gapdh* R CTCTTGCTCAGTGTCCTTGCTG	ThermoFisher	N/A
Mouse *Ifna4* F CCTGTGTGATGCAGGAACC	ThermoFisher	N/A
Mouse *Ifna4* R TCACCTCCCAGGCACAGA	ThermoFisher	N/A
Mouse *Ifnb1* F TCCACCAGCAGACAGTGTT	ThermoFisher	N/A
Mouse *Ifnb1* R CTTTGCACCCTCCAGTAATAGC	ThermoFisher	N/A
Mouse *Oas1* F AGTGTGGTGCCTTTGCCTGA	ThermoFisher	N/A
Mouse *Oas1* R GATGTCAAATCAGCCGTCAA	ThermoFisher	N/A
Mouse *Ifit3* F CATGCTGTAAGGATTCGCAAAC	ThermoFisher	N/A
Mouse *Ifit3* R GGGAAACTACGCCTGGATCTACT	ThermoFisher	N/A
Mouse *Cd69* F CCCTTGGGCTGTGTTAATAGTG	ThermoFisher	N/A
Mouse *Cd69* R AACTTCTCGTACAAGCCTGGG	ThermoFisher	N/A

**Software and algorithms**		

FACSDiva 6	BD Biosciences	RRID:SCR_001456
FlowJo 10	BD Biosciences	RRID:SCR_008520
Seurat v5.3.0	Hao et al.^51^	RRID:SCR_016341
STAR v2.7.10a	Dobin et al.^52^	RRID:SCR_004463
featureCounts v2.0.1	Liao et al.^53^	RRID:SCR_012919
DESeq2 v1.44.0	Love et al.^54^	RRID:SCR_015687
R v4.4.0	The R Foundation^55^	RRID:SCR_001905

### Experimental model and study participant details

#### Mice

C57BL/6J male and female mice (8–14 weeks old) were purchased from CLEA Japan (MGI:3055581), and sex- and age-matched mice were used for all experiments unless otherwise noted.

Transgenic mouse lines *Cxcr2*-CreERT2 (Accession No. IRCN-GE0057: https://core.ircn.jp/en/es-virus-core/)^56^ and *Icam1*-flox (Accession No. IRCN-GE0064; https://core.ircn.jp/en/es-virus-core/) were generated using a previously described genome engineering strategy.^57^

All mice were maintained under specific conventional conditions in a temperature- and humidity-controlled environment (23°C ± 3°C and 50 ± 20%) under a 12-h light/12-h dark cycle. Mice were housed in groups of up to six per cage with *ad libitum* access to food and water. Depending on the experiment, mice were housed either in Innocage (Innovive) or Micro BARRIER Systems (Edstrom Japan) containing PALSOFT wood-chip bedding (Oriental Yeast) and provided irradiated CE-2 chow (CLEA Japan), or in autoclaved polysulfone cages (CL-0123-3, CLEA Japan) within a B.B.H. Unit System (KE-2510, Theo Bit Co., Ltd.), containing autoclaved wood-chip bedding (Duo Rika Sangyo Co., Ltd.) and provided non-irradiated Lab MR Stock chow (Nosan Corporation).

Euthanasia was performed through isoflurane overdose followed by cervical dislocation. All animal procedures and experiments were approved by and performed in accordance with the guidelines of the Animal Care and Use Committee of the University of Tokyo (A2024P001) and Hokkaido University (2021-040).

#### Infections

Vesicular stomatitis virus (VSV) serotype New Jersey was inoculated intranasally in a 50 μL PBS volume under 5% isoflurane anesthesia. The viral dose was 1.0 × 10^8^ PFU for scRNA-seq and 1.0 × 10^7^ PFU for all other experiments unless otherwise specified. Mice that lost more than 30% of their initial body weight or survived longer than 14 days post-infection were euthanized in accordance with humane endpoint guidelines.

### Method details

#### Generation of *Icam1*-flox transgenic mouse lines

*Icam1*-flox: To construct the donor vector, a genomic fragment spanning 4,253 bp upstream of exon 1 through 3,042 bp downstream of exon 1 of the Icam1 locus was amplified by PCR from C57BL/6 mouse genomic DNA. Two *loxP* sites were inserted 30 bp upstream and 90 bp downstream of exon 1, respectively, to generate the floxed donor construct. The circular donor vector was co-transfected into C57BL/6-derived embryonic stem (ES) cells together with expression plasmids encoding SpCas9 and guide RNAs targeting 5′-GGGCAAAGTGCAGGTAGCAG-3′ and 5′-CTACGTGGGATTCGAACTCC-3′. Correctly targeted knock-in (KI) ES cell clones were identified and subsequently injected into eight-cell-stage ICR embryos. The embryos were cultured to the blastocyst stage and transferred into pseudopregnant ICR females. The resulting offspring, which were entirely derived from KI ES cells, were used as *Icam1*-flox mice for all experiments.

#### Tamoxifen-induced conditional deletion

*Cxcr2*-CreERT2 mice were crossed with *Icam1*-flox mice, and sex- and age-matched littermates (both male and female, 8–14 weeks old) were used to compare WT (*Cxcr2*^+/+^) and cKO (*Cxcr2*^CreERT2/+^) mice. All mice were homozygous for the floxed *Icam1* allele (*Icam1*^fl/fl^).

To induce Cre recombinase activity, mice received daily intraperitoneal (i.p.) injections of tamoxifen (100 mg/kg in corn oil) for five consecutive days, from 6 to 2 days before infection.

#### Blood and tissue sampling

150 μL of blood was collected from the tail artery into a tube containing 100 μL of 50 mM EDTA-2K in PBS. The red blood cells were lysed with ACK lysing buffer (Thermo, A1049201).

For the tissue dissection, mice were anesthetized with 200 μL of combination anesthetic (containing 75 μg/mL Medetomidine Hydrochloride, 400 μg/mL Midazolam, and 500 μg/mL Butorphanol Tartrate in saline). After perfusion with 10 mL of PBS, tissues including superficial cervical lymph nodes (scLNs), inguinal lymph nodes (iLNs), spleen, lung, and femoral bone marrow were collected. Cells were collected by mincing the lymph nodes through 35 μm nylon mesh. Bone marrow components were sampled by flushing the femur with 10 mL 1% Albumin, from Bovine Serum (BSA), Fatty Acid Free (Fujifilm, 011–15144) in HBSS(+) with 23G syringe.

For the biopsy of femur bone marrow cells, mice anesthetized with 200 μL of combination anesthetic and cells were aspirated with a syringe 27G needle inserted directly from knee into the femur. Mice were treated with 75 μg/mL Atipamezole Hydrochloride solution (200 μL) to antagonize the anesthesia.

#### ELISA

Whole blood samples in EDTA solution were centrifuged for 10 min at 1500 × g/4°C. The supernatants were collected as plasma samples and stored at −80°C. Concentrations of IFN-β (PBL Assay Science, No.42400-1) and corticosterone (Enzo Life Sciences, ADI-900-097) were determined by ELISA according to the manufacturer’s instructions.

#### Anti-PDCA1 antibody treatment *in vivo* to deplete plasmacytoid dendritic cell

Mice were treated with InVivoMAb anti-mouse CD317 (PDCA-1) (Bio X Cell, BE0311, 500 μg in 200 μL PBS, intravenously at the time of infection) or InVivoMAb rat IgG2b isotype control (Bio X cell, BE0090), following a previously described method.^20^

#### Clodronate liposome treatment *in vivo* to deplete subcapsular sinus (SCS) macrophages in scLNs

Mice were injected with clodronate liposomes into one side of the ear flap and control reagent into the contralateral side (HYG, 16003641; 50 μL per side), following a previously described method.^20^^,^^58^ Six days later, mice were subjected to infection.

#### Cytokine stimulation and dose selection

For *in vivo* IFN-β stimulation, recombinant mouse IFN-β1 (carrier-free) (BioLegend, 581306) diluted in 200 μL of 0.1% BSA/PBS was administered to uninfected mice by intravenous (i.v.) injection. Blood and bone marrow were collected 24 h after injection.

The IFN-β dose range was selected based on the estimated circulating IFN-β levels in our infection model and doses commonly used in previous *in vivo* studies.^59^^,^^60^ ELISA measurements indicated that plasma IFN-β levels in surviving mice could reach up to approximately 1600 pg/mL (Figure 3B), corresponding to an estimated circulating amount on the order of micrograms per mouse. Based on these considerations, 2.5 μg per mouse was used as a reference dose, and a 10-fold lower and higher dose (0.25, 2.5, and 25 μg per mouse) were tested together with a vehicle control.

#### Anti-IFNAR1 antibody treatment *in vivo* to inhibit type I IFN responses

Mice were treated with Anti-mouse IFNAR-1-InVivo (Selleck, A2121, 500 μg in 200 μL PBS i.p. at the time of infection, 1 dpi or 2 dpi) or Mouse IgG1 isotype control-InVivo (Selleck, A2106).

#### Zymosan administration *in vivo* to evaluate phagocytotic activity

Mice received injection of 100 μg Alexa Fluor 488 conjugated Zymosan A BioParticles (Invitrogen, Z23373) in 100 μL PBS at 1 dpi. The blood was sampled after 15 min and processed for flow cytometry analysis.

#### Flow cytometry

Collected cells were incubated at 4°C for 15 min with 4% paraformaldehyde (PFA) (Sigma-Aldrich, 441244). After centrifugation for 5 min at 800 × g/4°C, the cell suspensions were washed and resuspended in 3% BSA/PBS. The suspensions were incubated at 4°C for 30 min for surface staining. The list of flow antibodies is provided in key resources table. The samples were washed and resuspended in 0.2% BSA/PBS and filtered through a 35 μm mesh filter (Falcon). Flow cytometry data were acquired with FACS Aria III, IIIu (BD Biosciences) or CytoFLEX SRT (Beckman Coulter). Data was analyzed using FlowJo version 10 software. The following antibodies were used: PerCP/Cyanine5.5 anti-mouse CD45.2 clone 104 (1:200, Biolegend, 109828), APC/Cyanine7 anti-mouse CD11b clone M1/70 (1:200, Biolegend, 101226), Purified anti-mouse CD16/32 clone 93 (1:50, Biolegend, 101302), PE anti-mouse CD182(CXCR2) clone SA044G4 (1:200, Biolegend, 149304), APC anti-mouse CD54(ICAM1) clone YN1/1.7.4 (1:300, Biolegend, 116129).

#### Fluorescence-activated cell sorting (FACS)

Prior to sorting ICAM1^+^ and ICAM1^−^ neutrophils, cells were fixed with dithio-bis-succinimidyl propionate (DSP) (Thermo Scientfic, 22586) as previously described.^49^ This method inactivates the virus while preserving RNA integrity for downstream analysis. These fixed cells were then subjected to the staining protocol described above and were collected with using FACS Aria III or IIIu (BD Biosciences). Gating for sorting was applied using FACSDiva version 6.

#### Magnetic activated cell sorting (MACS)

For neutrophil isolation for bulk RNA-seq, blood-derived cells were incubated at 4°C for 15 min firstly with anti-CXCR2 antibody (1:50, clone SA044G4, BioLegend, 149304) and subsequently with anti-PE microbeads (1:100, Miltenyi Biotec, 130-048-801). Subsequently, cells were subjected to MACS (Miltenyi Biotec) and magnetically retained cells were eluted from the MS column and collected by centrifugation for 5 min at 400 × g/4°C.

#### Single-cell RNA sequencing and analysis

Blood was sampled at 0, 1 and 5 dpi as described in the previous section. Cells were fixed with CellCover (Anacyte, 800-125) at 4°C for 15min to preserve RNA. Cells were then resuspended in CELLBANKER 1 (Nippon Zenyaku Kogyo Co., Ltd., 11910) and stored at −80°C. After the final survival outcome was observed, samples from the survival (*n* = 2) and lethal (*n* = 2) groups were processed for subsequent library preparation following mRNA Whole Transcriptome Analysis (WTA) and Sample Tag Library Preparation Protocol from BD Rhapsody Single-Cell Analysis System with BD Mouse Immune Single-Cell Multiplexing Kit (BD, 633793). Sequencing was carried out on an HiSeq X Ten platform (Macrogen Japan) with paired-end 150 bp reads. Raw sequencing data were processed and analyzed using the BD Rhapsody WTA Pipeline, executed on the Seven Bridges Platform (Seven Bridges Genomics, Inc., https://www.sevenbridges.com). The pipeline included read alignment, UMI-based de-duplication, and generation of gene expression matrices based on BD’s reference transcriptome. In total, 1,710 single cells were sequenced: 166 and 208 cells at 0 dpi, 313 and 189 cells at 1 dpi, 515 and 319 cells at 5 dpi, from the survival and lethal groups, respectively. Cells expressing <200 features, >2,500 features, or >25% mitochondrial genes were excluded from the analysis. Dimensionality reduction and clustering analysis were performed using Seurat v5.3.0.^51^ The following cells were annotated using conventional cell markers: B cells (*Cd79a, Cd19, Ms4a*1), neutrophils (*Cxcr2, S100a8, S100a9*), Monocytes/DCs/Macrophages (*Cx3cr1, Csf1r, Adgre1*), T cells (*Cd3e*), NK cells (*Klrb1c, Nkg7*), and red blood cells (*Hba-a2*).

#### RNA extraction, reverse transcription, and qPCR

Total RNA was isolated from isolated cells or tissues of mice with the use of RNAiso Plus reagent (Takara, 9109) and ReverTra Ace qPCR Master Mix (Toyobo, FSQ301) following manufacturer’s protocols. The resulting cDNA was subjected to real-time PCR analysis in a LightCycler 96 instrument (Roche) with KAPA SYBR Fast qPCRKit (Kapa Biosystems). The amount of target mRNA was normalized by that of *Gapdh* mRNA.

**Table:** 

Primer name	Sequence (5′ to 3′)
Mouse *Gapdh* F	ATGAATACGGCTACAGCAACAGG
Mouse *Gapdh* R	CTCTTGCTCAGTGTCCTTGCTG
Mouse *Ifna4* F	CCTGTGTGATGCAGGAACC
Mouse *Ifna4* R	TCACCTCCCAGGCACAGA
Mouse *Ifnb1* F	TCCACCAGCAGACAGTGTT
Mouse *Ifnb1* R	CTTTGCACCCTCCAGTAATAGC
Mouse *Oas1a* F	AGTGTGGTGCCTTTGCCTGA
Mouse *Oas1a* R	GATGTCAAATCAGCCGTCAA
Mouse *Ifit3* F	CATGCTGTAAGGATTCGCAAAC
Mouse *Ifit3* R	GGGAAACTACGCCTGGATCTACT
Mouse *Cd69* F	CCCTTGGGCTGTGTTAATAGTG
Mouse *Cd69* R	AACTTCTCGTACAAGCCTGGG

### Quantification and statistical analysis

#### RNA sequencing analysis

Total RNA was isolated from the cells for library construction. RNA libraries were prepared using SMART-Seq Stranded Kit (Takara) following manufacturer’s protocols. Sequencing was carried out on an Illumina NextSeq 2000 platform with paired-end 36 bp reads or on a DNBSEQ-G400RS platform with 100 bp single-end reads. For DNBSEQ-G400RS sequencing, adaptor sequences were converted into the DNBSEQ format using the MGIEasy Universal Library Conversion Kit (App-A) (MGI). Sequences were mapped to the mouse reference genome (mm10) with the use of STAR v 2.7.10a.^52^ Raw read counts of each gene were calculated using featureCounts v2.0.^53^ Adjusted *p*-values and fold changes used to identify differentially expressed genes (DEGs) were calculated using the DESeq2 (v1.44.0) R package.^54^ Gene Ontology (GO) enrichment analysis for biological processes (BP) and Kyoto Encyclopedia of Genes and Genomes (KEGG) pathway enrichment analysis were conducted using the enrichGO and enrichKEGG functions in the clusterProfiler (v4.12.6) R package,^61^ with org.Mm.e.g.,.db and mouse genes (organism code: “mmu”) as annotation databases, respectively. Only terms and pathways with adjusted *p*-values <0.05 were considered significant. Plots were generated using ggplot within the ggplot2 package 3.5.2.^62^ The N1 and N2 signature genes shown in Figures 5C and S6A were selected based on previous studies.^63^^,^^64^^,^^65^^,^^66^^,^^67^

#### Statistics

All data were plotted and analyzed using R software (v.4.4.0) driving RStudio (v.2024.4.2.764). Data are presented as mean ± s.e.m. including the individual values where possible, and n represents the number of individual animals (biological replicates) unless otherwise noted. All flow cytometry images shown are representative of at least three independent experiments repeated with similar results. For comparisons between two groups, normality of the data distribution was assessed using the Shapiro–Wilk test, and data were considered normally distributed when *p* > 0.05. Means were compared using an unpaired two-tailed Welch’s *t* test if data were normally distributed, or a two-tailed Mann–Whitney U test if normality was not assumed (*p* ≤ 0.05). For paired comparisons, a paired two-tailed Student’s *t* test was used, since the differences between paired observations were confirmed to be normally distributed (*p* > 0.05). For comparisons among three or more groups, one-way ANOVA followed by Tukey’s post-hoc test was applied. When values were normalized to one condition (e.g., ICAM1^−^), a one-sample two-tailed *t* test was used to compare the normalized values to the hypothetical value of 1. Survival curves were generated using the Kaplan–Meier method and compared using the Wilcoxon (Breslow) test with BH correction for multiple comparisons. ∗∗∗∗*p* < 0.001; ∗∗∗*p* < 0.005; ∗∗*p* < 0.01; ∗*p* < 0.05; not significant (ns), *p* ≥ 0.05.
