# Supplementary material for: Type I IFN dynamics orchestrate protective ICAM1+ neutrophil heterogeneity as an early checkpoint for survival in lethal viral infection
Source: iScience. 2026 Aug 18;29(9):117189. doi: 10.1016/j.isci.2026.117189 (PMC13594436; doi:10.1016/j.isci.2026.117189)
Supplement: Supplementary file 1 [file mmc1.pdf]

iScience, Volume *29*

Supplemental information

Type I IFN dynamics orchestrate protective ICAM1^{+} neutrophil heterogeneity as an early checkpoint for survival in lethal viral infection

Riho Saito, Yuan Ding, Kureha Satou, Fumi Yamamoto, Akisawa Satomi, Hiroki Sugishita, Hideki Ukai, Yukiko Gotoh, and Tomohiko Okazaki

Figure S1

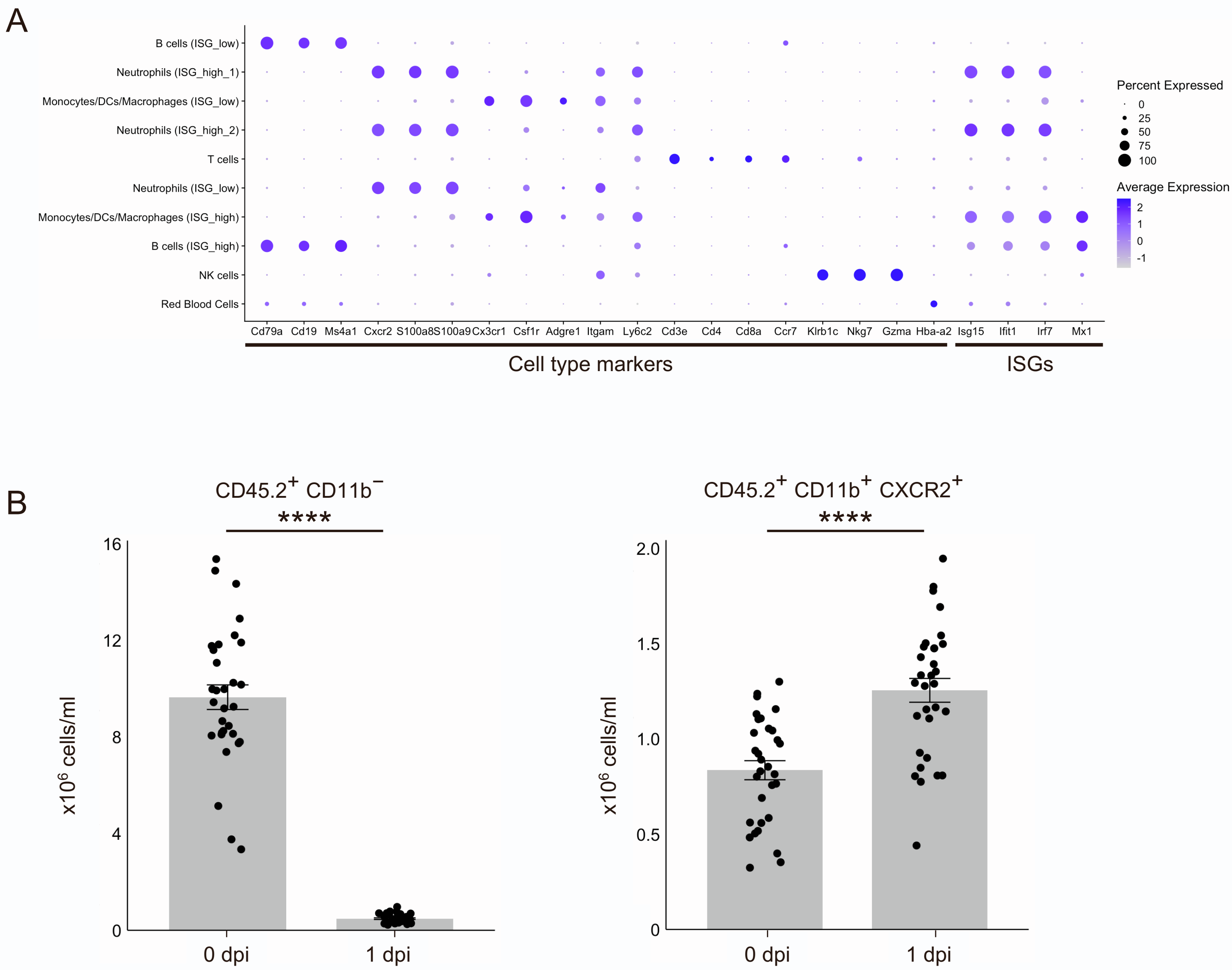

Figure S1. Expression profiles of canonical lineage markers and interferon-stimulated genes (ISGs) across all cell clusters, related to Figure 1.
(**A**) Dot plot showing the expression of canonical marker genes for cell type identification and key ISGs across all cell clusters identified by scRNA-seq.
(**B**) Flow cytometric analysis of absolute numbers of circulating $CD45^{+}CD11b^{-}$ lymphocytes and $CD45^{+}CD11b^{+}CXCR2^{+}$ neutrophils in male mice at 0 and 1 dpi (n = 31).

Data in (B) are presented as mean ± SEM. *****P* <0.001. Statistical significance was determined using paired, two-tailed Welch's t-test (B).

Figure S2

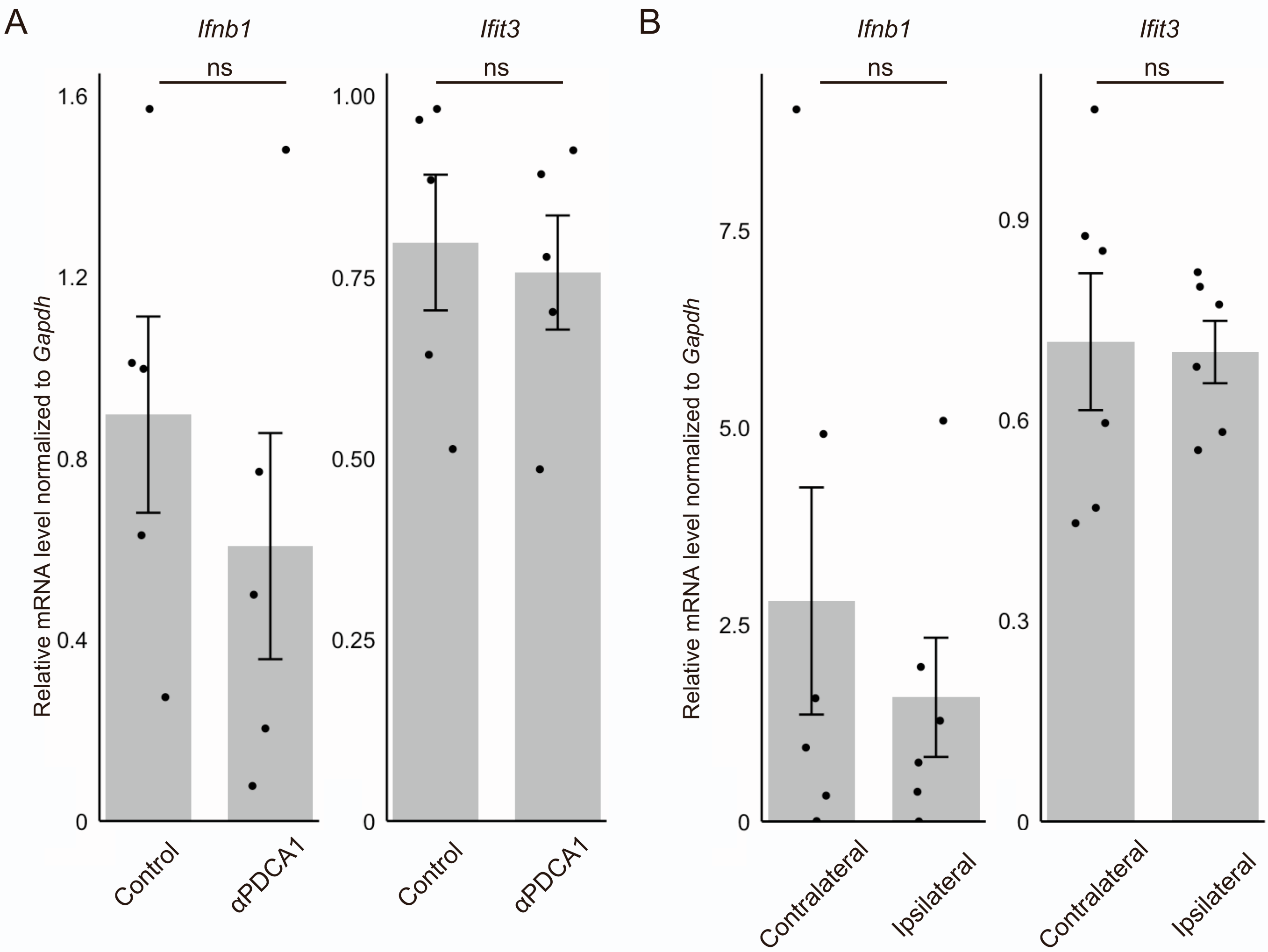

Figure S2. Effects of anti-PDCA1 antibody treatment and clodronate liposome injection on type I IFN signaling in draining lymph nodes.
(**A**) Relative mRNA levels of *Ifnb1* and *Ifit3* measured by RT–qPCR in superficial cervical lymph nodes (scLNs) at 1 dpi from male mice treated with anti-PDCA1 antibody for plasmacytoid dendritic cell (pDC) depletion or isotype IgG at 0 dpi (n = 5 per group).
(**B**) Relative mRNA levels of *Ifnb1* and *Ifit3* measured by RT–qPCR in scLNs at 1 dpi from male mice injected with clodronate liposomes on one side for depletion of subcapsular sinus (SCS) macrophages (ipsilateral) and with control liposomes on the contralateral side 6 days before infection (n = 6 mice).
Anti-PDCA1 neutralizing antibody treatment or clodronate liposome administration did not significantly change the expression levels.

Data are presented as mean ± SEM. ns, not significant. Statistical significance was determined using using an unpaired, two-tailed Welch’s t-test.

Figure S3

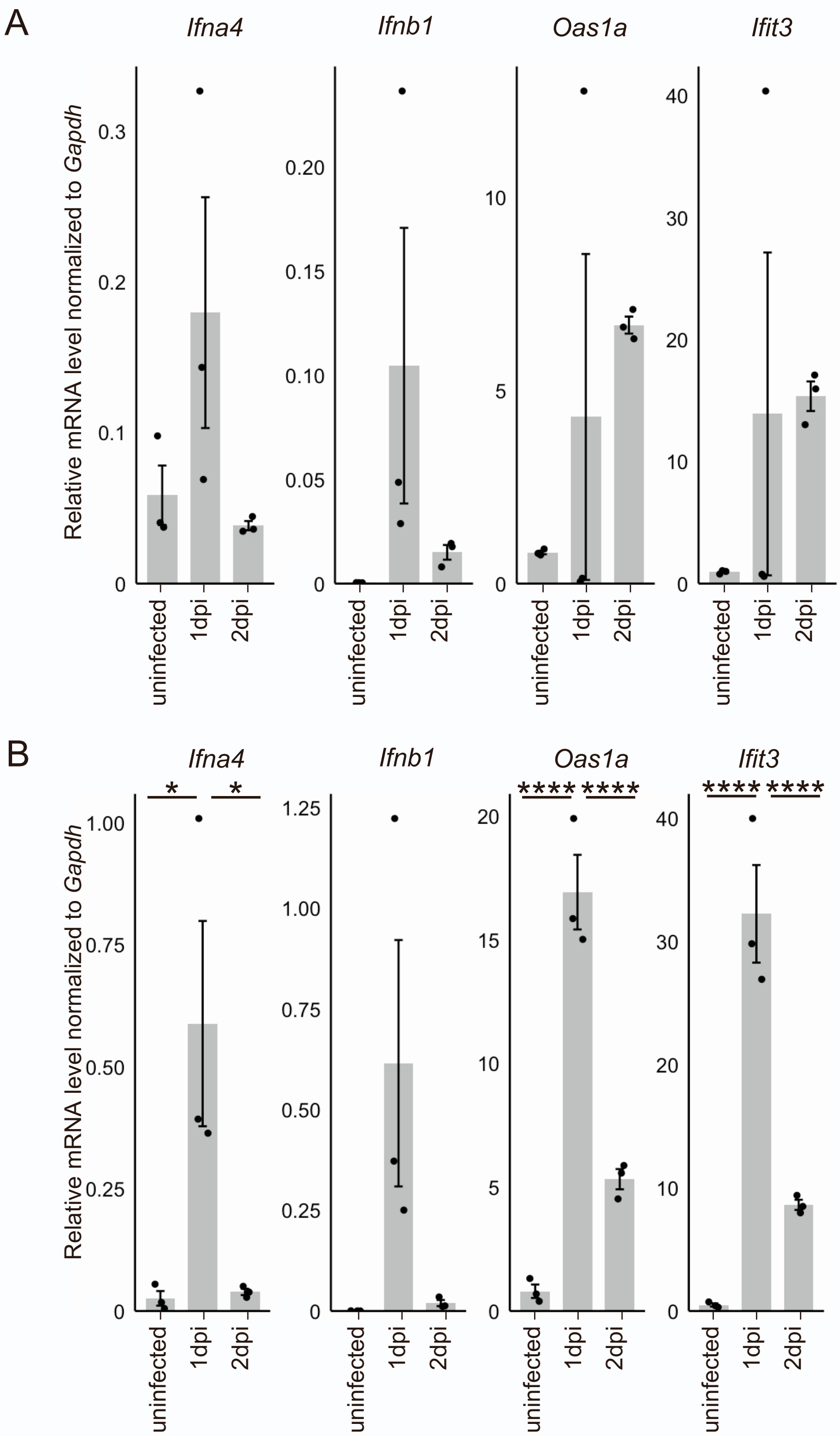

Figure S3. Expression levels of type I interferons and interferon-stimulated genes (ISGs) in lung and spleen.
Relative mRNA levels of *Ifna4*, *Ifnb1*, *Oas1a and Ifit3* measured by RT–qPCR in lung (**A**) and spleen (**B**) from uninfected and infected male mice at the indicated dpi (n = 3 each).

Data are presented as mean ± SEM. **P* <0.05. Statistical significance was determined using one-way ANOVA with Tukey's post-hoc test.

Figure S4

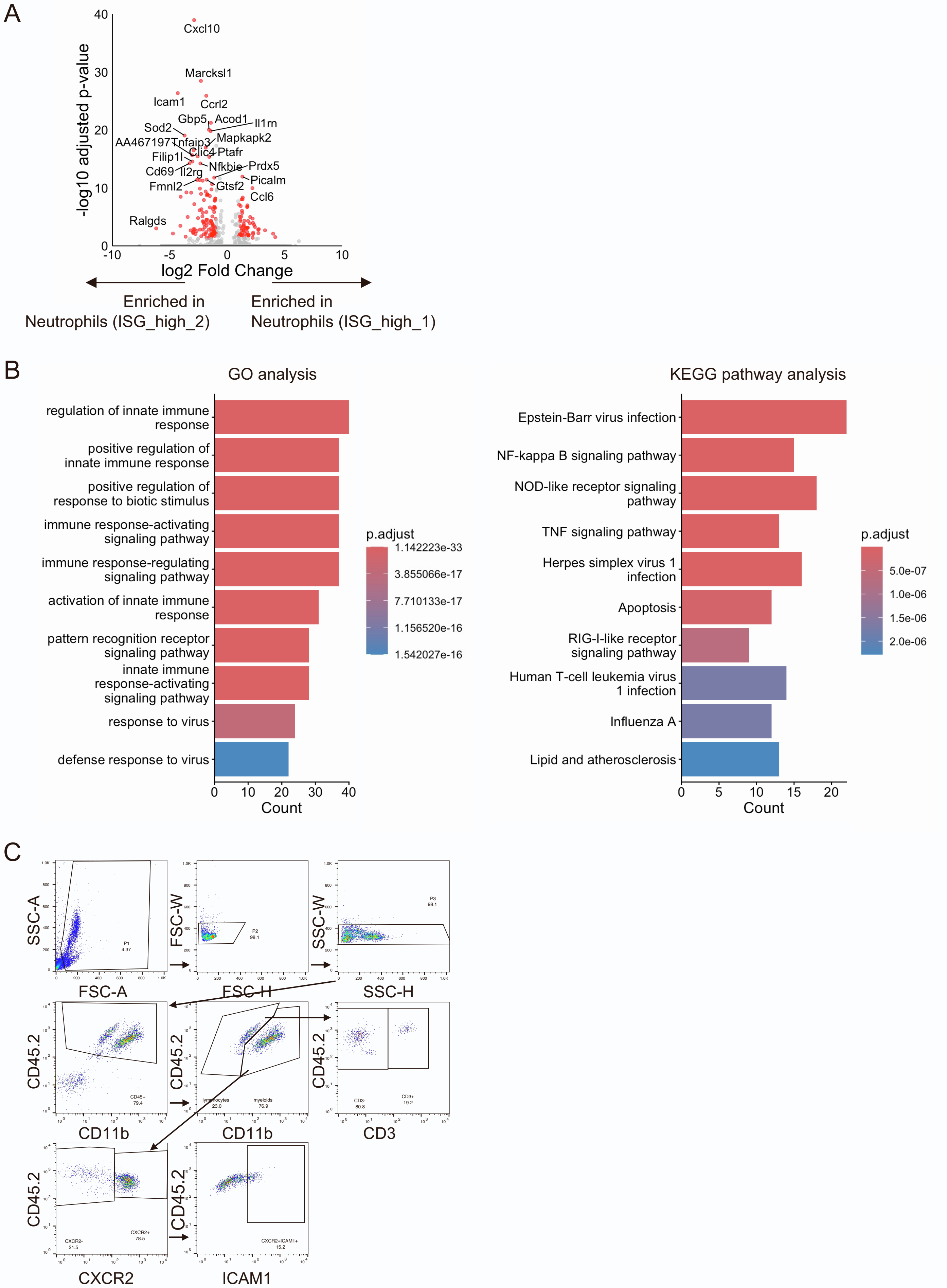

A
Cxcl10
Marcksl1
Icam1
Ccrl2
Sod2
Gbp5
Acod1
Il1rn
AA467197
Tnfaip3
Mapkapk2
Filip1l
Clic4
Ptafr
Cd69
Il2rg
Nfkbie
Prdx5
Picalm
Fmnl2
Gtsf2
Ccl6
Ralgds
-log10 adjusted p-value
log2 Fold Change
Enriched in Neutrophils (ISG_high_2)
Enriched in Neutrophils (ISG_high_1)
B
GO analysis
regulation of innate immune response
positive regulation of innate immune response
positive regulation of response to biotic stimulus
immune response-activating signaling pathway
immune response-regulating signaling pathway
activation of innate immune response
pattern recognition receptor signaling pathway
innate immune response-activating signaling pathway
response to virus
defense response to virus
Count
p.adjust
1.142223e-33
3.855066e-17
7.710133e-17
1.156520e-16
1.542027e-16
KEGG pathway analysis
Epstein-Barr virus infection
NF-kappa B signaling pathway
NOD-like receptor signaling pathway
TNF signaling pathway
Herpes simplex virus 1 infection
Apoptosis
RIG-I-like receptor signaling pathway
Human T-cell leukemia virus 1 infection
Influenza A
Lipid and atherosclerosis
Count
p.adjust
5.0e-07
1.0e-06
1.5e-06
2.0e-06
C
SSC-A
FSC-A
FSC-W
FSC-H
SSC-W
SSC-H
CD45.2
CD11b
CD11b
CD3
CXCR2
ICAM1

Figure S4. Distinct gene expression profiles between ISG-high neutrophil subsets, related to Figure 4.

(**A**) Volcano plot depicting DEGs between the scRNA-seq-defined Neutrophils (ISG_high_2) cluster and Neutrophils (ISG_high_1). Genes with an absolute log2 fold change > 1 and adjusted p-value < 0.05 are highlighted in red. (n = 3 mice)

(**B**) Gene Ontology (GO) and KEGG pathway analysis of 152 genes more highly expressed in the scRNA-seq-defined Neutrophils (ISG_high_2) cluster compared to the Neutrophils (ISG_high_1) cluster (adjusted p-value < 0.05, log2FoldChange < -1). The top 10 enriched terms are shown.

(**C**) Representative flow cytometry gating strategy for the identification of major immune cell populations in the blood

Figure S5

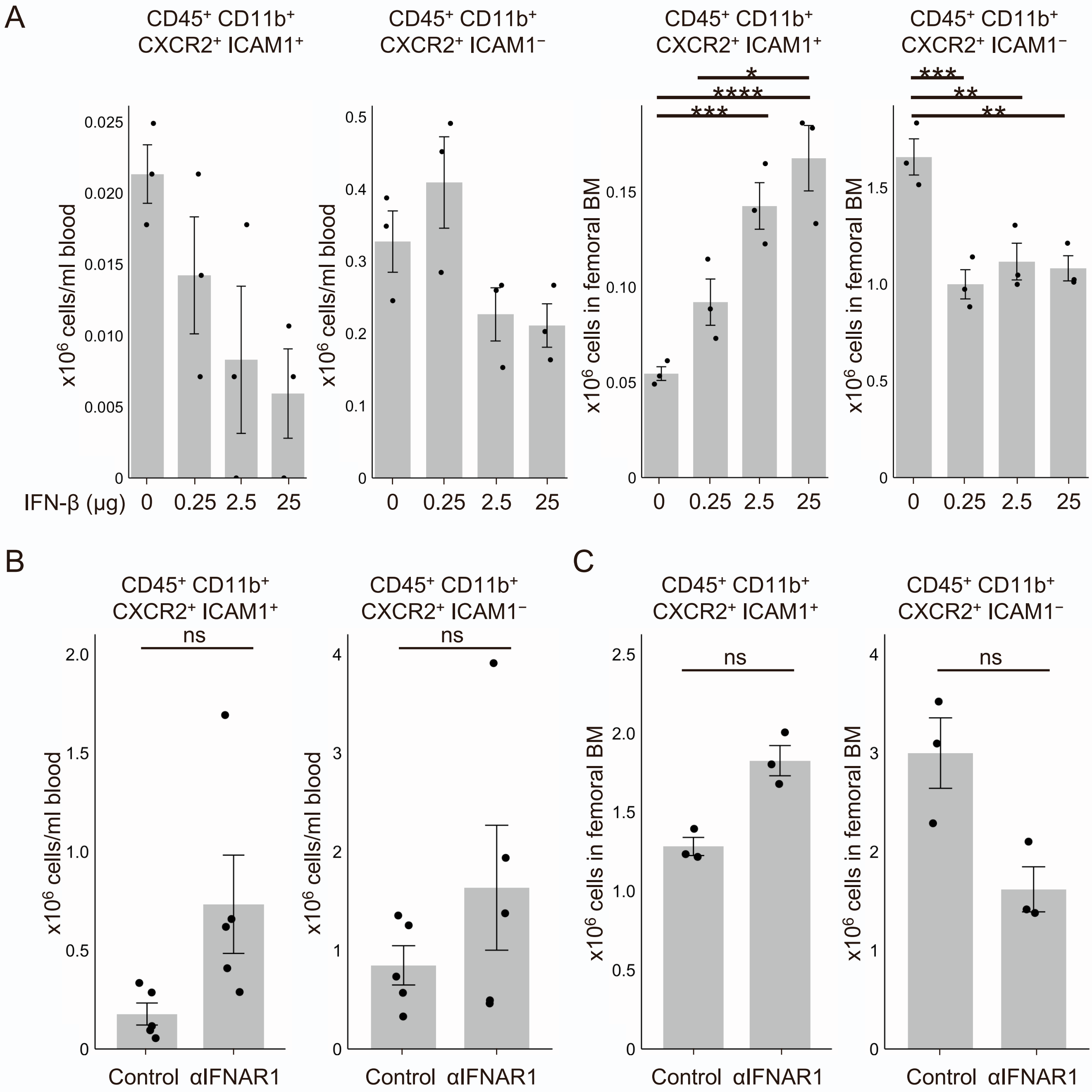

A
CD45⁺ CD11b⁺ CXCR2⁺ ICAM1⁺
CD45⁺ CD11b⁺ CXCR2⁺ ICAM1⁻
x10⁶ cells/ml blood
x10⁶ cells in femoral BM
IFN-β (μg)
0
0.25
2.5
25
B
C
ns
Control
αIFNAR1

Figure S5. IFNβ induced ICAM1 expression on CXCR2+ cells in the peripheral bone marrow (BM) but not in the blood, and type I IFN signaling blockade from 0 dpi did not markedly affect the number of ICAM1^{+} or ICAM1^{-} neutrophils at 1 dpi.

(**A**) Flow cytometric analysis of absolute numbers of CXCR2^{+} ICAM1^{+} and CXCR2^{+} ICAM1^{-} cells in the blood and femoral BM of female mice 24 h after i.v. administration of the indicated doses of IFN-β (n = 3 mice per group).

(**B**) Flow cytometric analysis of absolute numbers of CXCR2^{+} ICAM1^{+} and CXCR2^{+} ICAM1^{-} cells in the blood at 1 dpi in male mice treated with anti-IFNAR1 antibody or isotype IgG at 0 dpi (n = 5 per group).

(**C**) Flow cytometric analysis of absolute numbers of CXCR2^{+} ICAM1^{+} and CXCR2^{+} ICAM1^{-} cells in the femoral BM at 1 dpi in male mice treated with anti-IFNAR1 antibody or isotype IgG at 0 dpi (n = 3 per group).

IFNAR1 blockade led to a modest increase in ICAM1^{+} neutrophils in the blood and bone marrow, likely due to compensatory inflammatory signals (e.g., TNF-α) under high viral burden. Taken together, these results indicate that type I IFN signaling is sufficient to induce ICAM1^{+} neutrophils in the bone marrow, while additional inflammatory cues may also contribute to ICAM1 upregulation and are likely required for their systemic distribution.

Data are presented as mean ± SEM, where each point represents an individual mouse. ns, not significant, *$P < 0.05$, **$P < 0.01$, ***$P < 0.005$,****$P < 0.001$. Statistical significance was determined using one-way ANOVA with Tukey's post-hoc test (A), an unpaired, two-tailed Welch's t-test (B), or an unpaired, two-tailed Mann–Whitney U test (C). No significant differences were observed in the blood in (A).

Figure S6

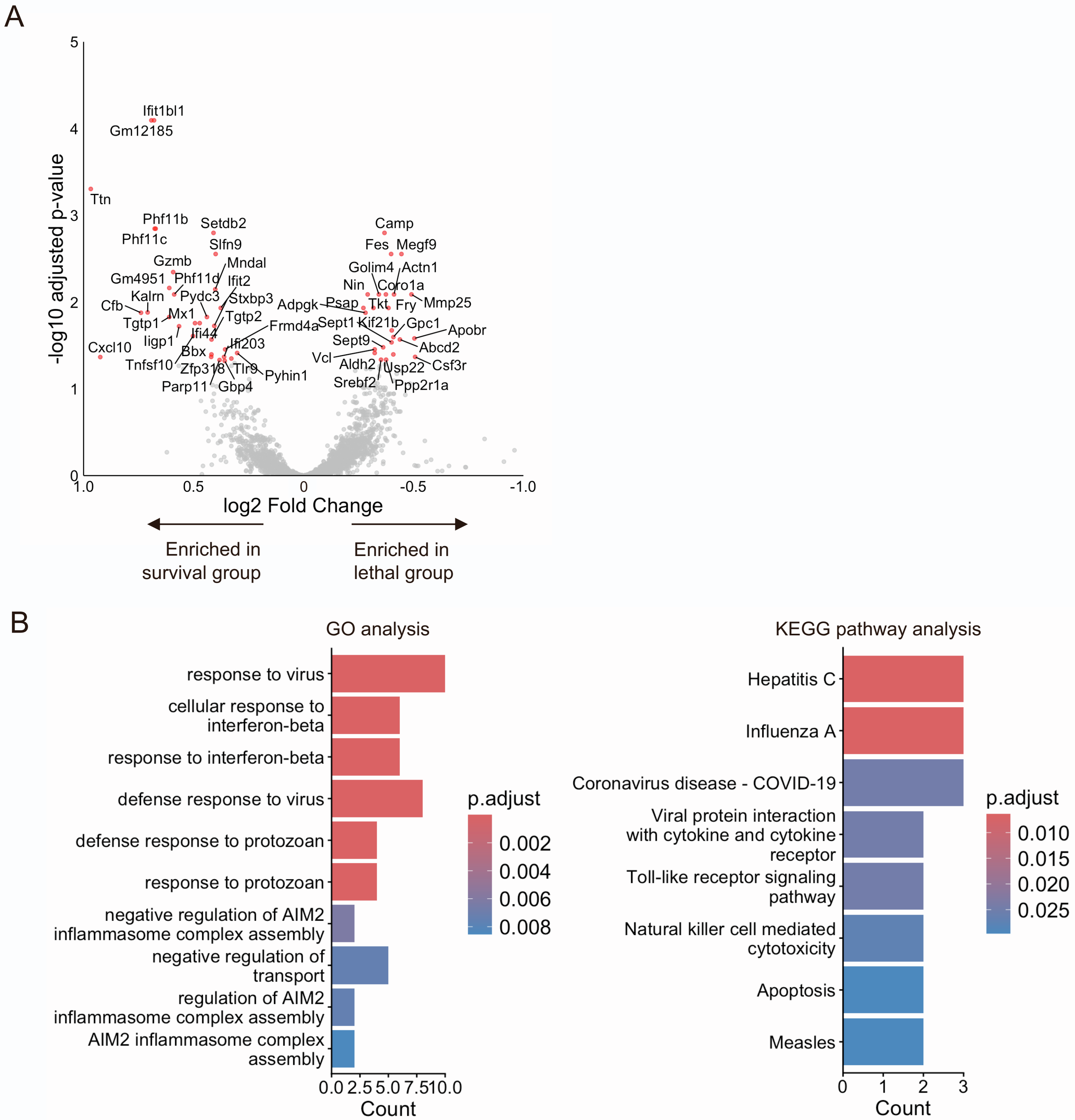

Figure S6. The survived group expressed higher levels of IFNβ signaling related genes in the bone marrow at 1 dpi.
(**A**) Volcano plot depicting DEGs identified by bulk RNA-seq of $CXCR2^+$ neutrophils in the femoral BM from male mice in the survived versus the lethal group (n = 6 each) at 1 dpi. Genes with an adjusted p-value < 0.05 are highlighted in red.
(**B**) Gene Ontology (GO) and KEGG pathway analysis of 31 genes more highly expressed in the femur BM from the survived group compared to the lethal group (adjusted p-value < 0.05, log2FoldChange < 0).

Figure S7

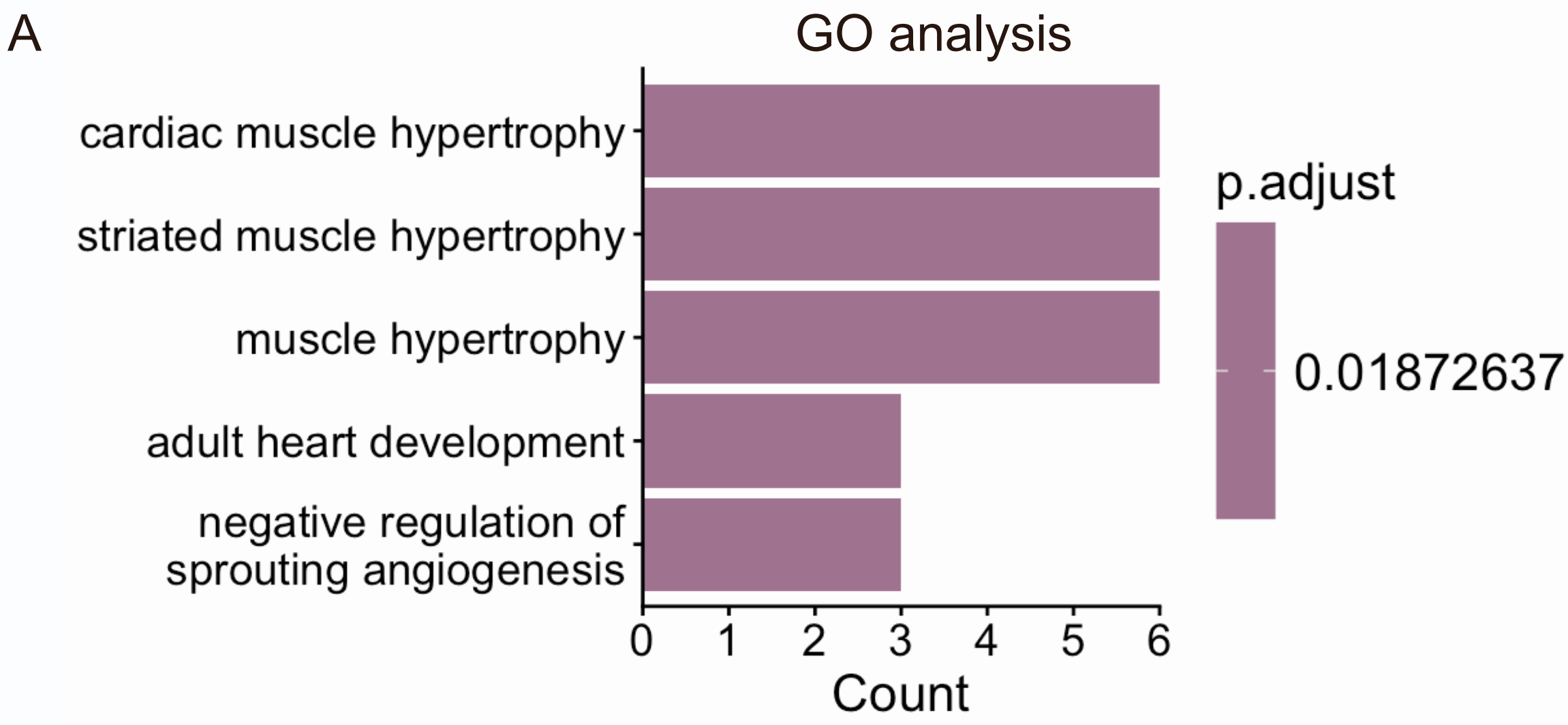

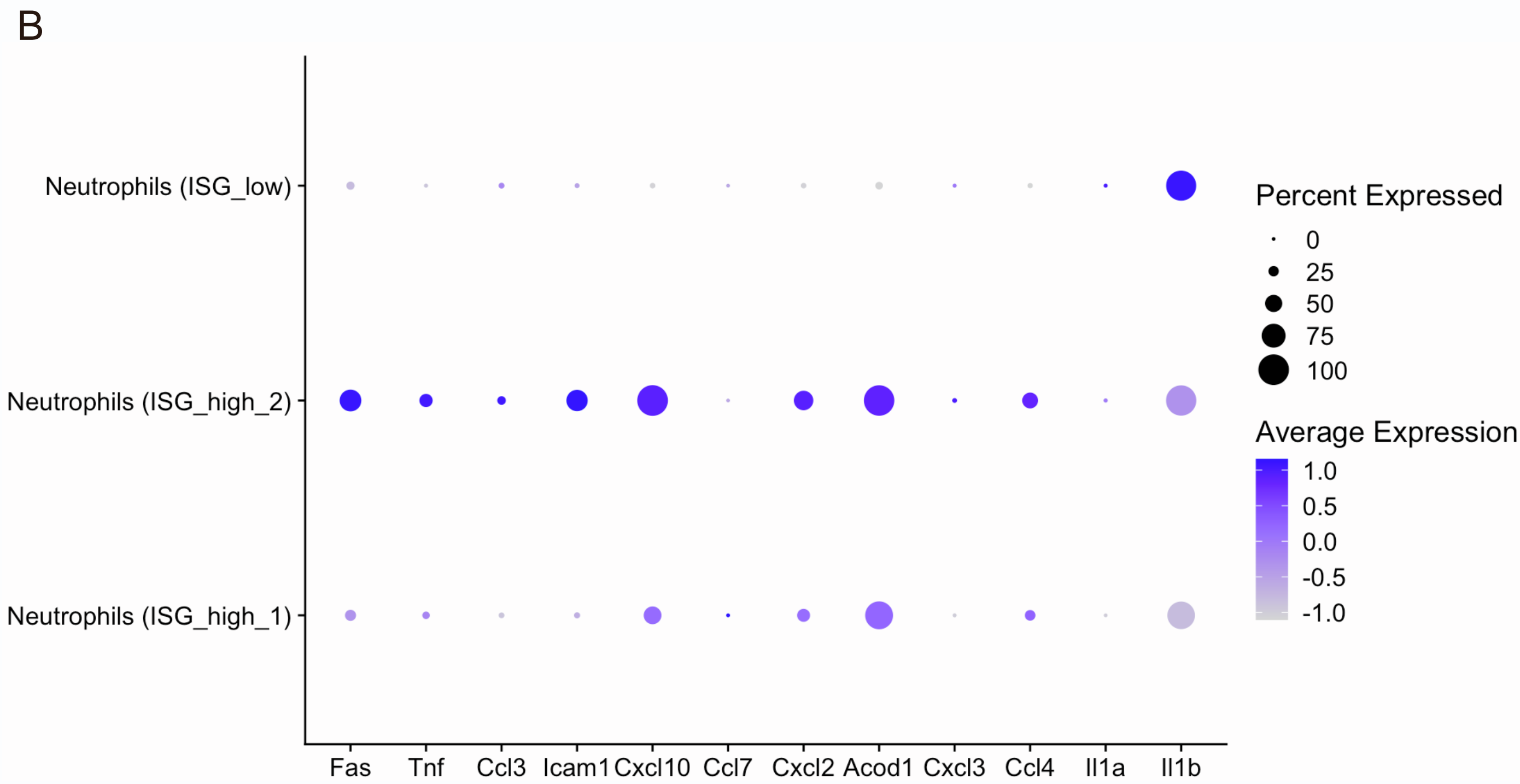

Figure S7. Additional characterization of circulating neutrophil subsets, related to Figure 5.

(**A**) Gene Ontology (GO) and KEGG pathway analysis of 149 genes more highly expressed in ICAM1^{-} neutrophils compared to ICAM1^{+} neutrophils (adjusted p-value < 0.05, log2FoldChange > 1). No enriched pathway was found in KEGG pathway analysis with this gene sets. (**B**) Dot plot from scRNA-seq data showing expression levels of N1 signature genes within each identified cell cluster.

Figure S8

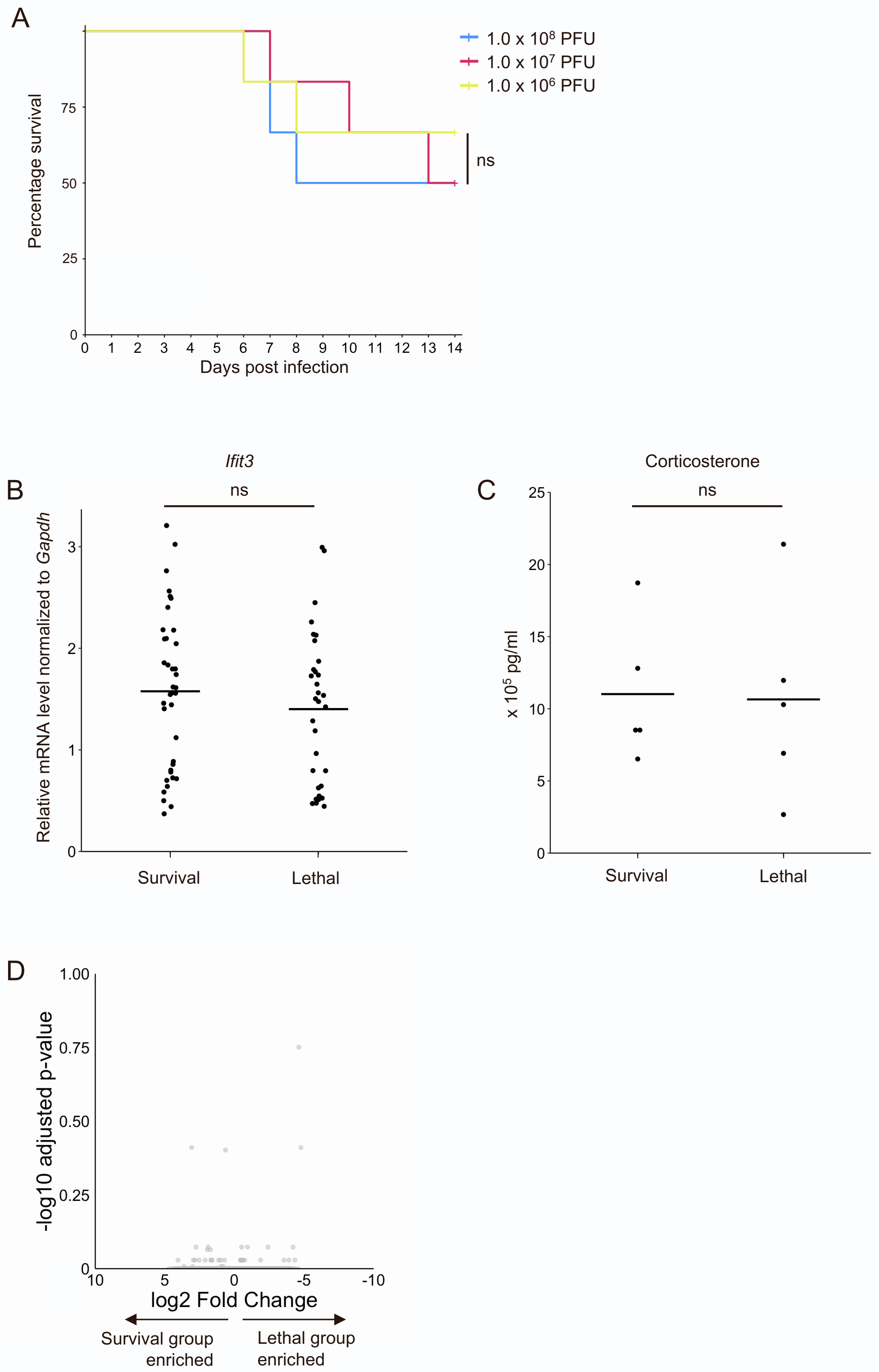

A
Percentage survival
75
50
25
0
0 1 2 3 4 5 6 7 8 9 10 11 12 13 14
Days post infection
1.0 x 10^8 PFU
1.0 x 10^7 PFU
1.0 x 10^6 PFU
ns
B
Ifit3
ns
Relative mRNA level normalized to Gapdh
3
2
1
0
Survival
Lethal
C
Corticosterone
ns
x 10^5 pg/ml
25
20
15
10
5
0
Survival
Lethal
D
-log10 adjusted p-value
1.00
0.75
0.50
0.25
0
10 5 0 -5 -10
log2 Fold Change
Survival group enriched
Lethal group enriched

Figure S8. No clear association was found between viral dose or baseline host factors and survival outcome

(**A**) Survival curves of female mice infected with the indicated doses of VSV (n = 6 each).

(**B**) Relative mRNA levels of *Ifit3* measured by RT–qPCR from blood circulating cells of male mice from survival and lethal groups before infection (n = 38 and n = 32, respectively).

(**C**) Corticosterone levels in the plasma of male mice in the survival and lethal groups before infection (n = 5 each).

(**D**) Volcano plot of RNA-seq analysis of circulating $CXCR2^+$ neutrophils from male mice that subsequently survived or succumbed to infection (n = 10 and 9, respectively), prior to infection (0 dpi). No differentially expressed genes (adjusted p-value < 0.05) were identified between the two groups.

Data in (B and C) are shown as a dot plot where each point represents an individual mouse and the horizontal line indicates the mean. ns, not significant. Statistical significance was determined using the Wilcoxon (Breslow) test with BH correction for multiple comparisons (A) or an unpaired, two-tailed Welch's t-test (B and C).

Figure S9

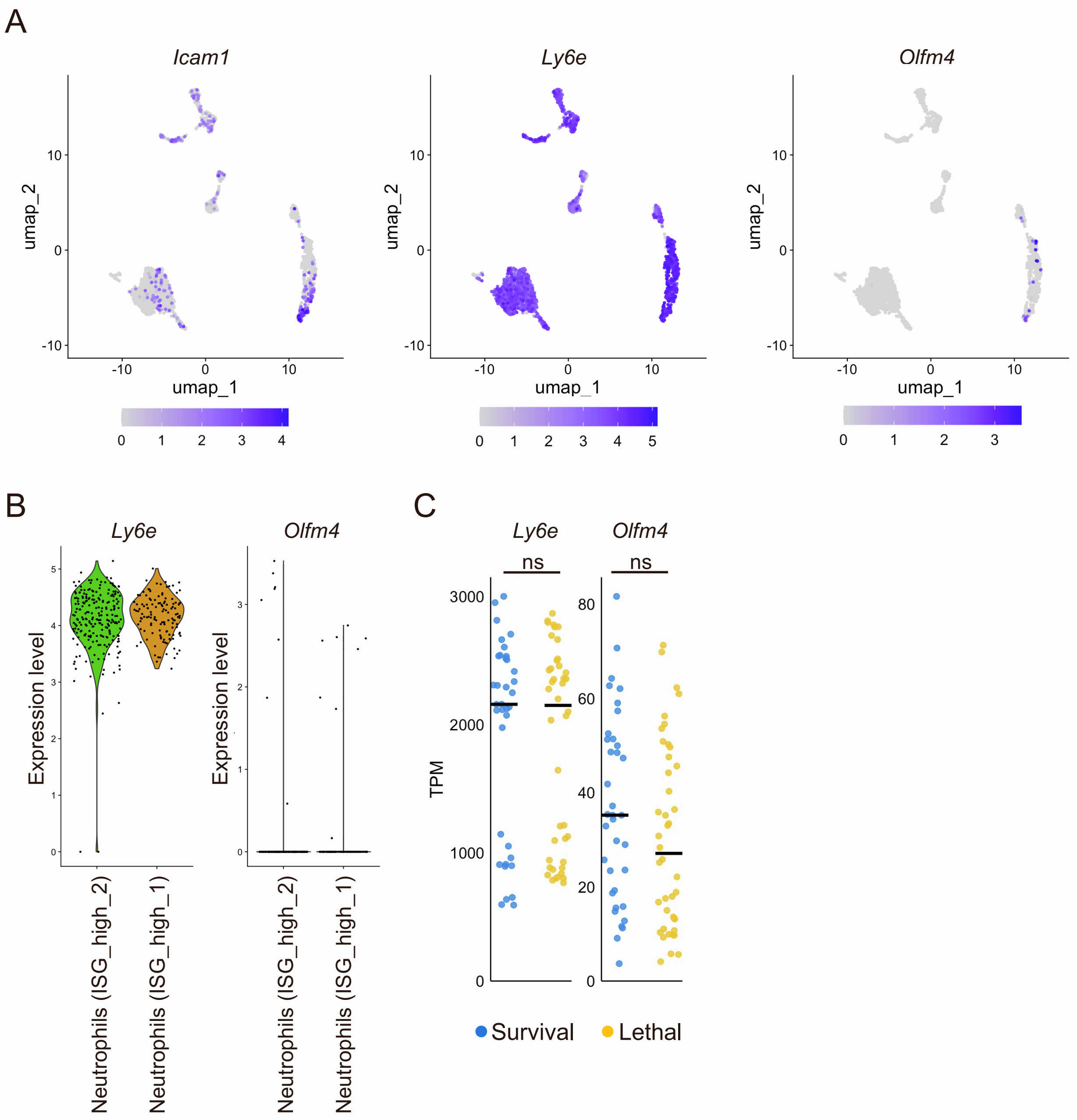

Figure S9. Neutrophil population identified in this study does not correspond to previously reported neutrophil subpopulations expressing Ly6e or Olfm4, related to Figure 4.

(**A**) Feature plots of the indicated genes from scRNA-seq analysis.

(**B**) Violin plots showing expression levels of the indicated genes in Neutrophils (ISG_high_1) and Neutrophils (ISG_high_2) clusters from scRNA-seq analysis.

(**C**) Expression levels of the indicated genes in sorted $CXCR2^{+}$ cells from bulk RNA-seq, comparing survival and lethal groups.

Data in (C) are presented as a dot plot, where each point represents an individual mouse and the horizontal line indicates the mean. ns, not significant. Statistical significance was determined using an unpaired, two-tailed Mann–Whitney U test.

Figure S10

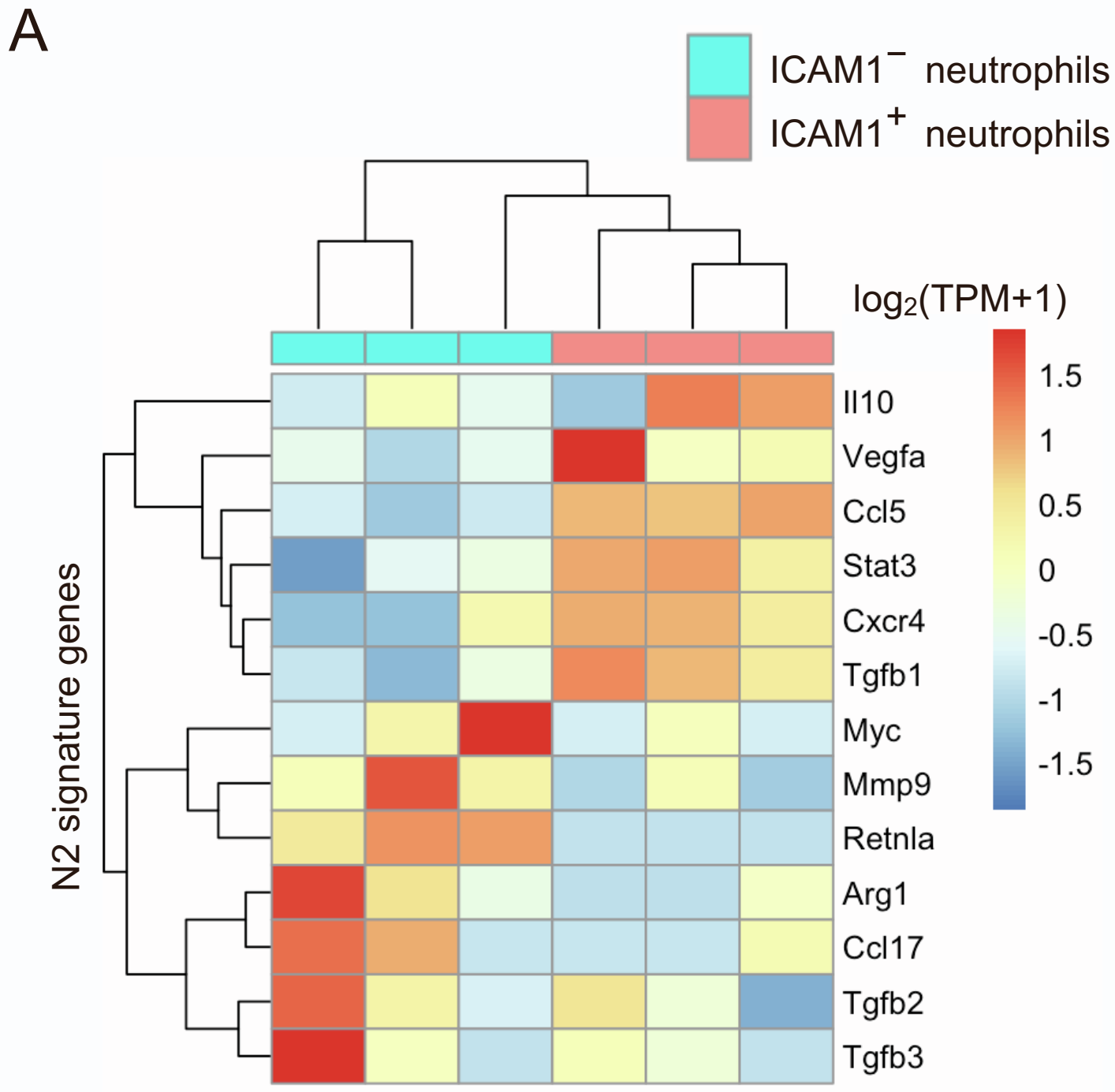

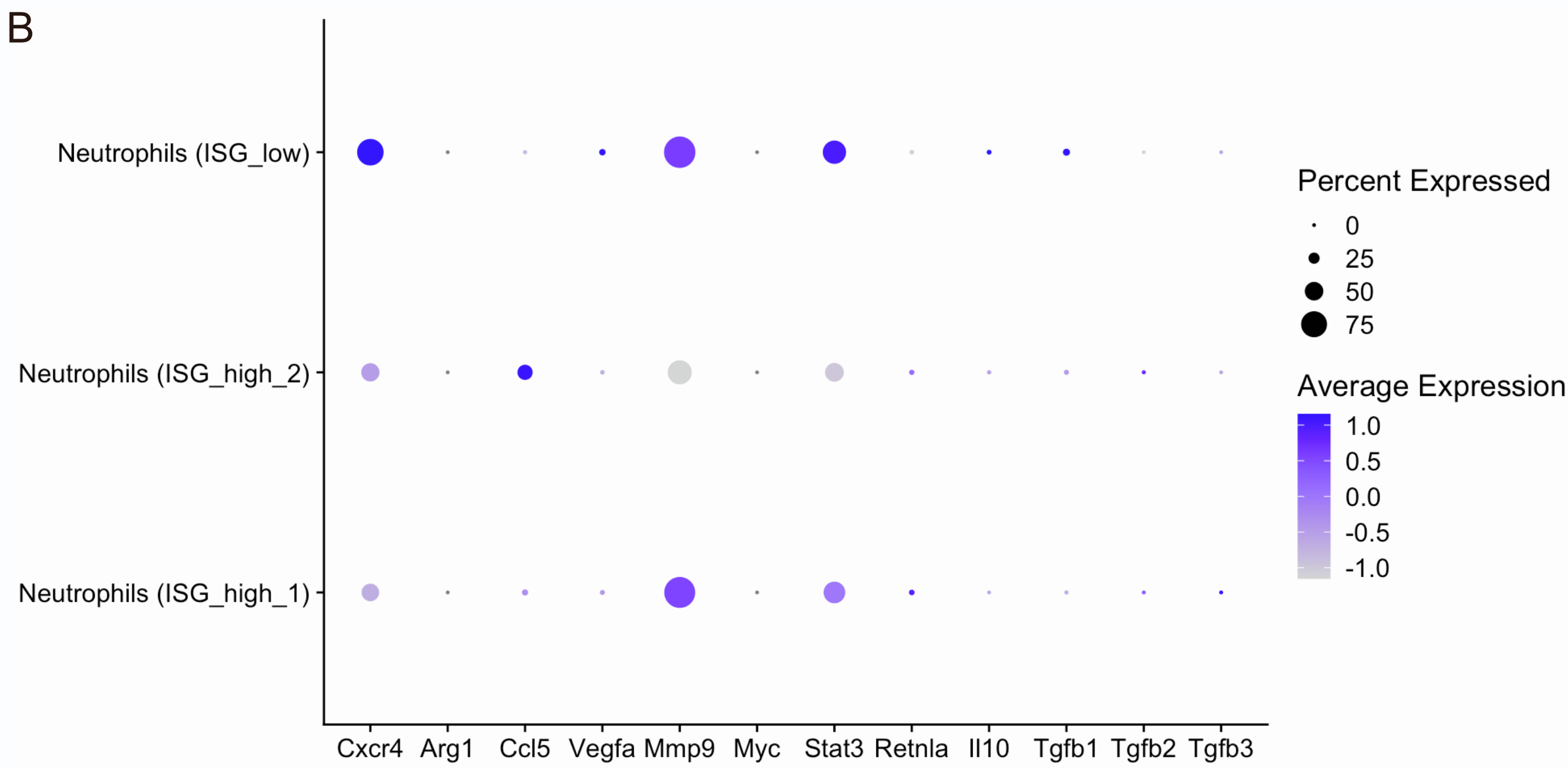

Figure S10. N2 signature genes are not specifically enriched in ICAM1⁻ neutrophils or the lethal group-associated ISG_high_1 cluster, related to Figure 5.
(A) Heatmap showing bulk RNA-seq expression of and N2 signature genes in sorted ICAM1⁻ and ICAM1⁺ neutrophils.
(**B**) Dot plot from scRNA-seq analysis showing expression levels of N2 signature genes within each identified cell cluster.
